# Loss of stomach, loss of appetite? Sequencing of the ballan wrasse (*Labrus bergylta*) genome and intestinal transcriptomic profiling illuminate the evolution of loss of stomach function in fish

**DOI:** 10.1186/s12864-018-4570-8

**Published:** 2018-03-06

**Authors:** Kai K. Lie, Ole K. Tørresen, Monica Hongrø Solbakken, Ivar Rønnestad, Ave Tooming-Klunderud, Alexander J. Nederbragt, Sissel Jentoft, Øystein Sæle

**Affiliations:** 1 Institute of Marine Research, P.O. Box. 1870, Nordnes, 5817 Bergen, NO Norway; 2 Centre for Ecological and Evolutionary Synthesis, Department of Biosciences, University of Oslo, P.O. Box 1066, Blindern, 0316 Oslo, NO Norway; 3 Department of Biology, University of Bergen, P.O. Box 7803, 5020 Bergen, NO Norway; 4 Biomedical Informatics Research Group, Department of Informatics, University of Oslo, P.O. Box 1066, Blindern, 0316 Oslo, Norway

**Keywords:** Ballan wrasse, Stomach, Intestine, Ghrelin, Labrid, Digestion, Motilin, Transcobalamin, Appetite, Neuropeptide

## Abstract

**Background:**

The ballan wrasse (*Labrus bergylta*) belongs to a large teleost family containing more than 600 species showing several unique evolutionary traits such as lack of stomach and hermaphroditism. Agastric fish are found throughout the teleost phylogeny, in quite diverse and unrelated lineages, indicating stomach loss has occurred independently multiple times in the course of evolution. By assembling the ballan wrasse genome and transcriptome we aimed to determine the genetic basis for its digestive system function and appetite regulation. Among other, this knowledge will aid the formulation of aquaculture diets that meet the nutritional needs of agastric species.

**Results:**

Long and short read sequencing technologies were combined to generate a ballan wrasse genome of 805 Mbp. Analysis of the genome and transcriptome assemblies confirmed the absence of genes that code for proteins involved in gastric function. The gene coding for the appetite stimulating protein ghrelin was also absent in wrasse. Gene synteny mapping identified several appetite-controlling genes and their paralogs previously undescribed in fish. Transcriptome profiling along the length of the intestine found a declining expression gradient from the anterior to the posterior, and a distinct expression profile in the hind gut.

**Conclusions:**

We showed gene loss has occurred for all known genes related to stomach function in the ballan wrasse, while the remaining functions of the digestive tract appear intact. The results also show appetite control in ballan wrasse has undergone substantial changes. The loss of ghrelin suggests that other genes, such as motilin, may play a ghrelin like role. The wrasse genome offers novel insight in to the evolutionary traits of this large family. As the stomach plays a major role in protein digestion, the lack of genes related to stomach digestion in wrasse suggests it requires formulated diets with higher levels of readily digestible protein than those for gastric species.

**Electronic supplementary material:**

The online version of this article (10.1186/s12864-018-4570-8) contains supplementary material, which is available to authorized users.

## Background

The wrasses belong to one of the largest teleost families, the Labridae, with 600 species and 82 genera [1]. Most labroids are small colorful predators living on invertebrates that they pick off the substrate with their protruding teeth. Several wrasse species occupy an important niche in the ecosystem as cleaner fish, consuming ectoparasites from the skin and gills of other fish species. The ballan wrasse (*Labrus bergylta*) is the largest of the European wrasses and is found in the eastern Atlantic, from mid-Norway in the north to the southwest region around Morocco, the Azores and Madeira and southeast into the Mediterranean ocean [2]. In captivity, ballan wrasse display cleaning behavior when introduced to sea lice-infected salmon [3]. Thus, the wrasses, including the ballan wrasse, have been used as an alternative to chemicals and pharmaceuticals for delousing salmon in net pens [4], and attempts are underway to rear ballan wrasse for this purpose.

In addition to its peculiar reproductive strategies and feeding behaviors, the wrasses all lack stomachs (i.e. agastric) and pyloric caeca, and have a very short intestine. Agastric species are found sporadically throughout the teleost lineage in unrelated species. Other examples of agastric fish includes the fugu *(Takifugu rubripes)*, green spotted pufferfish *(Tetraodon nigroviridis)*, zebrafish *(Danio rerio)* and medaka *(Oryzias latipes)* [5]. Comparative genetic analyses demonstrates that loss of stomach function is accompanied with multiple gene losses related to acid production [5]. However, fundamental questions on how and why these past evolutionary losses have occurred, and the functional implications for agastric species remain unknown. Furthermore, there is a need for basic physiological and genetic knowledge of wrasses to enable the formulation of diets that meet the nutritional requirements of this and other agastric species in aquaculture.

The stomach is an anterior expansion of the gut separated from the intestine by a sphincter, i.e. a valve, at the distal end regulating the release of food to the intestine. The stomach varies greatly in shape, from tubular to asymmetric, between different fish species [6]. It serves a range of physiological purposes, mainly related to digestion. These include: 1) storage and retention of ingested food, permitting the ingestion of fewer and larger meals (macrophagous species) [7, 8], 2) release of hydrochloric acid to aid digestion [9], 3) digestion of proteins by the release of enzymes, 4) providing a steady supply of partly digested feed (chyme) to the anterior mid-gut to optimize digestion and absorption, 5) lowering pH to act as a barrier against pathogens, 6) facilitating absorption of several micronutrients, e.g. Fe^3+^, Ca^2+^, and B_12_, and 7) playing a role in osmo- and ion regulation [9]. How these digestive functions are performed by agastric species remains unknown. So far, there is no coherent hypothesis that merges the characteristics of agastric animals [7]. The digestive system in agastric fishes is thus very diverse, we therefore hypothesize that specific knowledge on the molecular regulation of digestion and appetite in wrasses cannot be addressed with existing data from the existing agastric species genomes.

Advances in high throughput sequencing now allows the analysis of how genetic drift or evolutionary forces change genomes through deletion or inactivation of genes when an organ is acquired or lost, while maintaining a continuation of an animals overall function. Our main aim was to investigate the genetic impact of stomach loss on the digestive functions in ballan wrasse with a special focus on appetite regulation. Here, we first describe the sequencing and assembly of the ballan wrasse genome and two de novo transcriptomes, allowing us to search for genes related to stomach function and to identify key genes involved in appetite regulation. We also generated expression profiles along the length of the wrasse gastrointestinal tract to identify possible functional organization. We found that wrasse lack nearly all the genes related specifically to stomach digestion, and the gene for vertebrate appetite control, ghrelin. Many genes related to intestinal tract digestion were highly expressed in the anterior segments of the wrasse intestine, with decreasing expression of these genes occurring towards the intestine posterior. The data presented in this study provides valuable insight into the genomics underlying the functional adaptations to an agastric digestive system, and should aid the formulation of diets better suited to wrasse aquaculture.

## Results

### Genome assembly statistics and annotation

The ballan wrasse genome was based on integration of long and short sequencing reads, i.e. from PacBio (33 x coverage) and Illumina (111 x coverage) respectively. The N50 contig length of 704 kbp (Table 1) surpasses the size of many of the recently sequenced teleost genomes; yellow croaker (63.11 kbp, [10]), northern pike (16.91 kbp, [11]) and Atlantic cod (116 kbp) [12, 13]. and it is defined as the shortest contig length at 50% of the genome. The N50 contig length approaches the N50 contig length of the Asian seabass sequenced to 90X PacBio coverage at 1 Mbp [14]. The N50 scaffold length is 795 kbp (Table 1), suggesting that the mate pairs utilized had marginally more information than the PacBio reads. This means that the PacBio reads covers most of the same repeats as for the illumina mate pairs. The N50 contig length is actually equal to the N50 scaffold length obtained for northern pike. This shows that medium coverage of PacBio sequences can generate assemblies of comparable or superior N50 statistics to other teleost genome assemblies based on short-read data. The N50 statistics constitute the length that 50% of the genome contigs surpass. Moreover, the completeness of the ballan wrasse genome, measured as number of conserved eukaryotic genes using tools that estimate gene set completeness, CEGMA and BUSCO, was, for instance, comparable to the newest version of the Atlantic cod genome [12, 13].

**Table 1:** Genome assembly statistics

	Ballan wrasse statistics
Total assembly length (Mbp)	805
N50 scaffold (kbp)	795
N50 contig (kbp)	704
CEGMA complete (partial)	92.34% (94.35%)
BUSCO complete (% of 3698)	3368 (91%)
BUSCO duplicated (% of 3698)	155 (4.2%)
BUSCO fragmented (% of 3698)	110 (3.0%)
BUSCO missing (% of 3698)	65 (1.8%)

Approximately 27 Gbp of PacBio sequencing data (34 X coverage) were assembled with canu [15], and the resulting contigs were ordered and oriented into scaffolds with Illumina data using the scaffold module of SGA [16], before polishing with Pilon [17]. The resulting ballan wrasse genome assembly has a total size of 805 Mbp, with an N50 contig length of 704 kbp and an N50 scaffold length of 795 kbp (Table 1).

The automatic annotation tool MAKER2 [18, 19] annotated 87,438 gene models, which were reduced to 31,050 after removing gene models that were poorly supported by either transcriptome or protein alignments. To simplify, gene models with less than half of their bases supported by an alignment were removed).

### De novo wrasse Illumina transcriptome assembly statistics and annotation

De novo RNA assemblies were generated using Illumina short read sequence reads containing brain and intestinal tissue sequences. The overall transcriptome assembly statistics were improved following RSEM filtering, reducing the number of assembled transcripts (Trinity genes) from 174,273 to 41,235 and increasing the N50 length from 2699 to 2794 (Additional file 1). In total, 26,618 genes (44%) of the transcripts were annotated with BLASTx (*p* < 1.0E-20) against the Uniprot database. Of these, 7730 cover more than 90% of the best Uniprot hit protein length, indicating full-length sequences (Additional file 2).

### Search for stomach function related genes and neuropeptides in the ballan wrasse

To identify genes involved in stomach function retained in ballan wrasse, we searched for genes expressed in the human stomach that were previously identified through transcriptomic profiling of the human gastrointestinal tract [20] (Table 2). We also genes related to appetite regulation. We first conducted BLAST searches (BLASTn and BLASTx) against the ballan wrasse genome and the de novo wrasse Illumina transcriptome assembly presented in this manuscript (Flow chart can be viewed in Additional file 3) using transcripts representing orthologue genes from the closely related species as query sequences. If a matching sequence was identified in the ballan wrasse genome or transcriptome, these ballan wrasse sequences was as used as a BLAST query sequence against the NCBI (https://blast.ncbi.nlm.nih.gov/Blast.cgi) non redundant protein sequences database (nr) database (BLASTx) and the nucleotide collection (nr and nt) database (BLASTn). If the investigated stomach target gene were not among the top hits of the previous mentioned BLAST search the gene was finally declared not present in ballan wrasse. If we got a strong hit, we used gene synteny looking at neighboring gene to validate the finding. The synteny analysis would reveal if this was the investigated target gene or if it was a closely related paralogous gene. We also conducted synteny searches looking for the target genes in specific genomic regions with shared neighboring genes [21].

**Table 2:** Presence (+) and absence (−) of genes related to stomach function and appetite regulation ballan wrasse

Function			Mammals	Ballan Wrasse
Stomach function	PG	Pepsinogen genes	+	–
	ATP4A	ATPase H+/K+ Transporting Alpha Subunit	+	–
	ATP4B	ATPase H+/K+ Transporting Beta Subunit	+	–
	GAST	Gastrin	+	–
	LIPF	Lipase F, Gastric Type	+	–
	TFF1	TFF1	+	–
	PGC	Progastricin	+	–
	MUC5AC	Mucin 5 AC, Oligomeric Mucus/Gel-Forming	+	+
	GIF	gastric intrinsic factor	+	–
Apetite regulation	GHRL	ghrelin	+	–
	GHRS	ghrelin receptor	+	+
	MBOAT4	membrane Bound O-Acyltransferase Domain Containing 4	+	–
	MNL	motilin	+	+
	MLNR	motilin receptor	+	+
	PYY	peptide yy	+	+
	CCK	cholecystokinin	+	+
	NPY	Neuropeptide y	+	+
	POMC	proopiomelanocortin	+	+
	LEP	Leptin	+	+

#### Presence and absence of genes related to stomach function

Using the above mentioned search strategy, the only stomach related gene found in the ballan wrasse genome was *muc5ac* (LABE_00062109) (Table 2). The *muc5ac* gene was also not present in the intestinal/brain transcriptome. cBLAST searches and synteny analysis confirmed the absence in wrasse of several stomach related genes, including gastrin (gast), lipase F (lipf), TFF1, pepsinogens (pgc), *atp4*, *atp4b*, previously described as being absent in other agastric fish [5] verifying the absence of stomach function and related genes in wrasse.

#### Presence and absence of genes related to ghrelin

We also searched the wrasse genome for the presence/absence of ghrelin (*ghrl*), ghrelin receptor (*ghsr*) and membrane bound o-acyltransferase domain containing 4 (*mboat4*) since ghrelin is connected to stomach function and appetite regulation in gastric species and produced in the gastric folds of both mammals and teleosts [22].

**Fig. 1:**
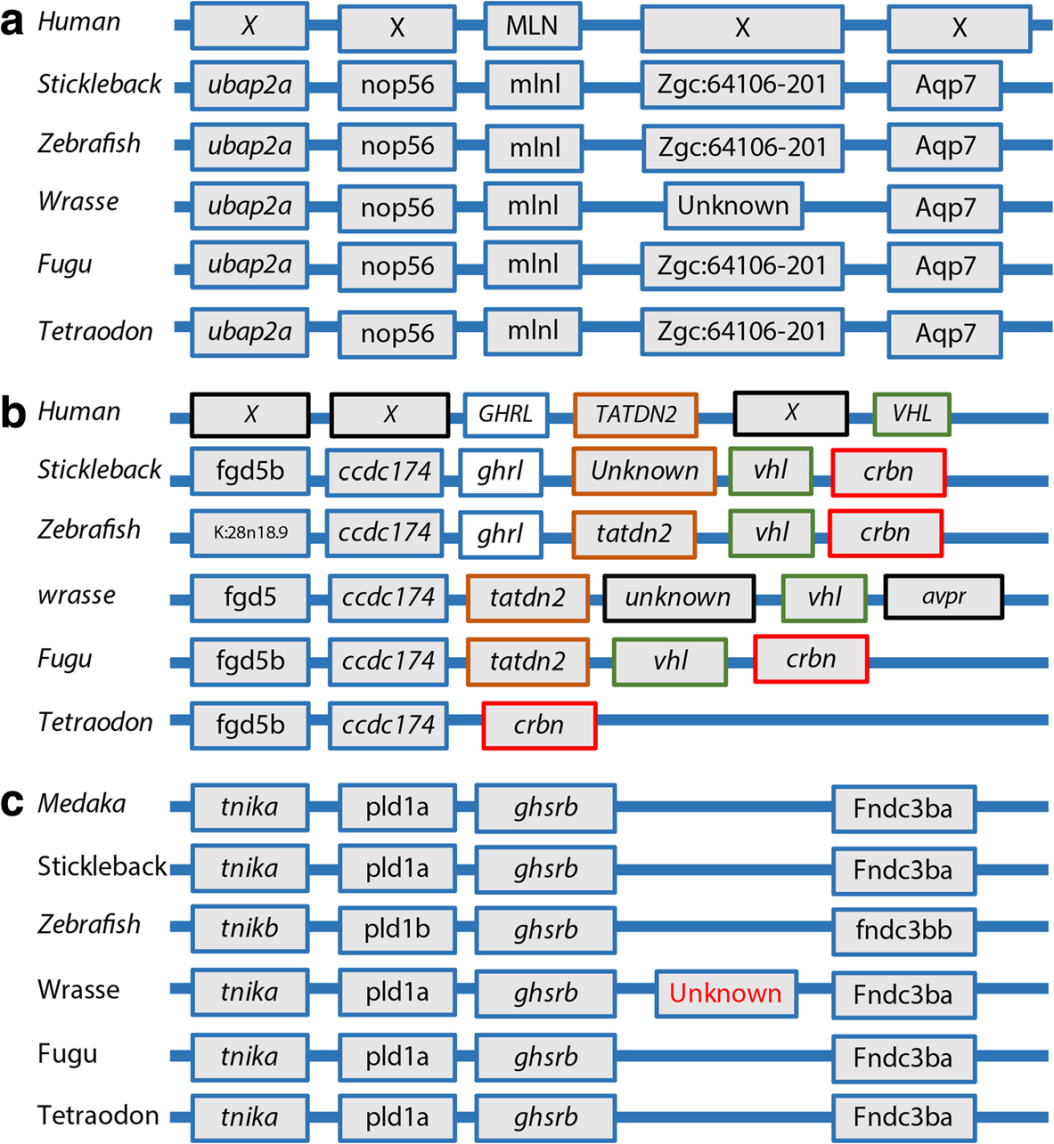
Genomic region and flanking genes for **a**) ghrelin (*ghrl*) in teleosts and in humans. Human (*Homo sapiens*): Chromosome 3:10164509–10,710,322, ENSG00000157017; Stickleback (*Gasterosteus aculeatus*): scaffold_27:2056484–2,136,261, ENSGACG00000000762; zebrafish (*Danio rerio*): Chromosome 6:40415935–40,469,646, ENSDARG00000054239; Ballan wrasse (*Labrus bergylta*): 160104_scaffold_265:308430–378,850; Fugu (*Takifugu rubripes*): scaffold_190:396861–429,246; Tetraodon (*Tetraodon nigroviridis*): 11:7296715–7,322,780. **b** Genomic region and flanking genes for ghrelin receptor b (ghsrb) gene in teleosts: Medaka (*Oryzias latipes*): Chromosome 22:4877644–5,093,878, ENSORLG00000011709; Stickleback (*Gasterosteus aculeatus*): groupXV:10000952–10,147,608, ENSGACG00000010956; Zebrafish (*Danio rerio*): Chromosome 24:26345585–26,748,381, ENSDARG00000057117; Ballon wrasse (*Labrus bergylta*): LaB_20160104_scaffold_576:107441–261,984, LABE_00056989; Fugu (*Takifugu rubripes*): scaffold_158:423578–529,307, ENSTRUG00000012927; Tetraodon (*Tetraodon nigroviridis*): chromosome 10:9436035–9,503,906, ENSTNIG00000006665. **c** Genomic region and flanking genes for motilin (mln) for human (*Homo sapiens*) and motilin like (mlnl) for teleosts. Human (6:33721662–33,933,669), Stickleback (*Gasterosteus aculeatus*) (groupXVII:8418961–8,450,006), Zebrafish (21:11822889–11,931,100), Ballan wrasse (*Labrus bergylta*) (LaB_20160104_scaffold_265:345347–348,493), fugu (*Takifugu rubripes*) (scaffold_116:360717–388,624) and tetraodon (*Tetraodon nigroviridis*) (11:1616728–1,638,376). The figure is based on genetic information collected form the Ensembl database release 84 for all species except for Ballan wrasse (current release). All accession numbers are from the Ensembl (http://www.ensembl.org/) database except for B. wrasse. Coloring of boxes have been added to make it easier for the reader to see the differences in synteny between the teleosts

Ghrelin has been identified in several cyprinid fish species including zebrafish (Ensembl accession no. ENSDARG00000054239), horned golden-line barbell (*Sinocyclocheilus rhinocerous,* NCBI accession no. XP_016371398.1) and goldfish (*Carassius auratus*) [23], as well as in several gastric species, for example in stickleback (*Gasterosteus aculeatus*) (NCBI accession no. KT235779). BLAST searches in the Ensembl database using ghrelin sequences from zebrafish and stickleback showed no similarity against any sequence of searched agastric fish species including ballan wrasse, amazon molly *(Poecilia formosa*), medaka, fugu and tetraodon. In addition, no ghrelin like putative coding sequence in the genomic region beween *ccdc174* and *tatdn2* in ballan wrasse (160104_scaffold_265:340,771–355,031) could be found (Fig. 1a). By searching the Ensembl database in the region between the genes *ccdc174* and *tatdn2,* we confirmed that among agastric fish species, the ghrelin gene is only found in the cyprinid family. The membrane bound o-acyltransferase domain containing 4 (*mboat4*) was not found in wrasse using *mboat4* (ENSGACT00000018180) of stickleback as a template for BLASTX and tBLASTn searches against the de novo transcriptomes and genome, or by analyzing gene synteny. In Ensembl, *mboat4* teleost orthologues were found only for stickleback, cave fish and zebrafish. BLAST searches were then used on the genomes of several gastric fish species represented in the Ensemble database, which confirmed *mboat4* is also present in tilapia, spotted gar (*Lepisosteus oculatus*) and Atlantic cod (*Gadus morhua*) among others. In these fish, *mboat4* is situated in a conserved genomic region (synteny shown in Additional file 4: Figure SA). Zebrafish aside, no orthologs of *mboat4* were identified in the genomes of agastric fish species present in Ensembl.

We found a gene coding for the ghrelin receptor (*ghsrb*) (LABE_00056989) in the genomic region (LaB_20160104_scaffold_576:107441–261,984) of wrasse, and that *ghsrb* was highly conserved among teleosts (Fig. 1b).

#### Presence and absence of genes coding for neuropeptides related to digestion and appetite

We then searched the genome and transcriptomes for expression of genes related to neuropeptides (other than ghrelin) involved in regulation of digestion and appetite, including motilin (*mln*), motilin receptor (*mlnr*), peptide yy (*pyy*), cholecystokinin (*cck*), neuropeptide y (*npy*) and proopiomelanocortin (*pomc*). Neuropeptides expressed in vertebrate brains and gastrointestinal tracts are thought to play key roles in digestion and appetite control. Our local gene synteny analyses identified motilin in *ballan wrasse* (Fig. 1c). The deduced ballan wrasse motilin amino acid sequence (LABE_00007846) aligned closely with the motilin protein of other fish species [24], further supported the existence of motilin in ballan wrasse. A ballan wrasse motilin receptor was also identified (LABE_00040338), neighboring *fndc3a*, the same region as in other fish species (Additional file 4: Figure SB).

Two genes coding for the peptide YY (*pyy*) were found, *pyya* (LABE_00046718) and *pyyb* (LABE_00049925) in the wrasse genome using gene synteny established from other species (Additional file 4: Figures SC & SD). Some variation occurred in the genes neighboring *pyya* and *pyyb*, but overall, most teleosts showed an overall synteny within the region containing these genes. No *pyyb* gene was identified in fugu or tetraodon. The automatic annotation in the current study annotated LABE_00022765 as *pyy*, but manual BLAST searches and synteny analyses identified this gene as an *npy* orthologue. Two *cck* like genes, similar to *cckb* (LABE_00034484) and *ccka* (LABE_00001570) were identified in wrasse (synteny shown in Additional file 4: Figures SE & SF). Two forms of leptin (*lep*); *lepa* (LABE_00052950) and *lepb* (LABE_00004184); were also identified in the wrasse genome.

### Transcriptional profiling of the ballan wrasse intestine

**Fig. 2:**
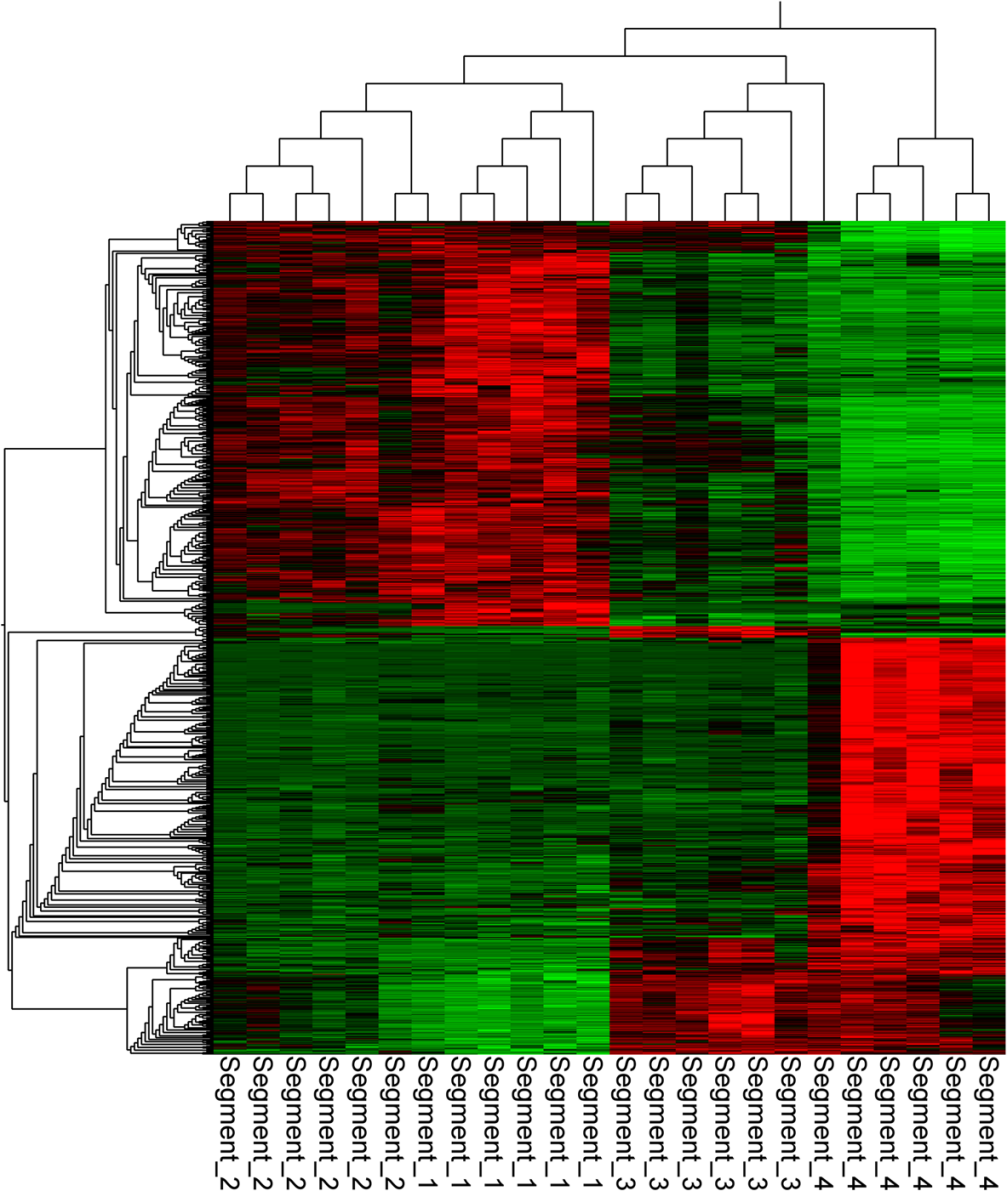
Hierarchical clustering of the 1000 most significantly differentially expressed genes (q < 1.6942e-4) separating the four intestinal segments. The figure was generated using multi group comparison implemented in the Qluecore omics explorer software. Arrow; genes uniquely enriched in segment 3 but not in segment four

**Fig. 3:**
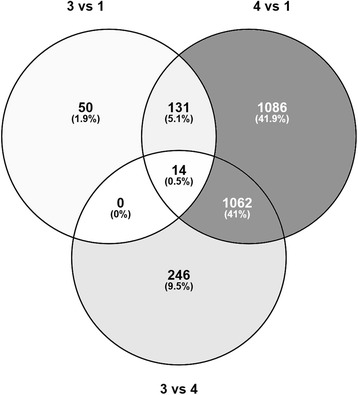
Venn diagram showing overlapping and non overlapping genes in segment 1, 4 and 3

RNAseq analysis of the four sequential intestinal segments (*n* = 6) that together encompassed the entire length of the wrasse intestine (see Additional file 5) revealed a total of 4853 differentially expressed genes (q < 0.05) between the segments (Additional file 6). Hierarchical clustering of the 1000 most significantly differentially expressed genes, and the much larger number of differentially expressed transcripts, between segment 4 and intestinal segments 1–3 demonstrates segment 4 (hind-gut) differs substantially from the rest of the intestine (Fig. 2 and Table 3). Meanwhile few differences were found between segments 1, 2 and 3. Fifty genes were identified as uniquely enriched in segment 3 (Fig. 3), but no significantly enriched pathways were identified within this list using DAVID functional annotation tool (FDR < 5%).

**Table 3:** Differentially expressed genes between segments 1–4 (p-adjust < 0.05)

*Gene regulation*	*1* vs *2*	*2* vs *3*	*3* vs *4*	*1* vs *4*
*Up*	11	39	990	2119
*Down*	3	37	1322	2293
*Total*	14	76	2312	4412

**Fig. 4:**
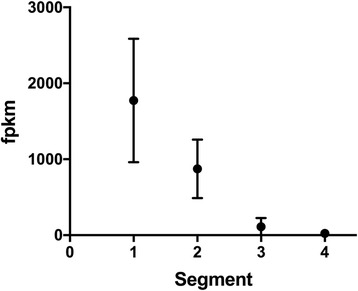
Gene expression levels of transmembrane protease serine 15 (*tmprss15*) in four segments along the wrasse intestine from anterior to posterior. The expression values are normalized RNAseq read counts expressed as fragments pr kilobase of transcript per million (fpkm)

Genes involved in general nutrient digestion and uptake of proteins were highly expressed in the anterior part of the intestine. In general, there was a declining expression gradient, anterior to posterior, for most genes involved in protein and fatty acid digestion and uptake, as exemplified by transmembrane protease serine 15 (*tmprss15*) (Fig. 4). Both of the Solute Carrier Family 15 Member 1 (*slc15A1,* aka *pept1*) gene paralogs (a and b) were more abundantly expressed in segment 1 compared to segment 4, while *slc15A2* (aka *pept2*) was more highly expressed in segment 4 (Additional file 6).

Differentially expressed genes were further analyzed using the DAVID Bioinformatics Resources 6.8 (Beta) to identify possible functional fingerprints for the intestinal segments.

Functional analysis using DAVID resulted in a large list of enriched (FDR < 5%) GO terms (Additional file 7) in addition to enriched KEGG pathways for segment 1 (Table 4) and segment 4 (Table 5). The GO terms reported by DAVID were further clustered using REVIGO to obtain an overview of the main enriched biological functions. The analysis shows that gene expression in the anterior part of the intestine is dominated by metabolism of lipid, glycerophospholipid, cellular protein and lipoprotein, as well as cholesterol homeostasis (Additional file 8). The posterior segment (segment 4) was dominated by genes involved in DNA replication, mitotic cell cycling, selenium compound metabolism, cell division and transferrin transport. Within the latter cluster, we found several terms related to endocytosis including “endocytosis”, “endosomal transport” and “intercellular protein transport”. As the gene expression levels were similar between segments 1 and 2, and only two functional terms related to bile acid secretion were enriched in segment 3 vs 2 only enriched GO terms between segment 1 and 4 are shown.

**Table 4:** Kegg pathways enriched in segment 1 versus 4 (FDR < 5%)

Term	Gene Count	FDR %
hsa01100:Metabolic pathways	210	2.5E-14
hsa01200:Carbon metabolism	35	2.8E-06
hsa01130:Biosynthesis of antibiotics	51	1.0E-05
hsa04146:Peroxisome	27	1.1E-04
hsa00564:Glycerophospholipid metabolism	29	1.2E-04
hsa04975:Fat digestion and absorption	17	5.7E-04
hsa00020:Citrate cycle (TCA cycle)	14	0.003
hsa00260:Glycine, serine and threonine metabolism	16	0.005
hsa00280:Valine, leucine and isoleucine degradation	17	0.011
hsa04976:Bile secretion	21	0.013
hsa03320:PPAR signaling pathway	20	0.030
hsa00640:Propanoate metabolism	12	0.041
hsa00071:Fatty acid degradation	15	0.091
hsa04931:Insulin resistance	25	0.212
hsa04923:Regulation of lipolysis in adipocytes	16	0.437
hsa00620:Pyruvate metabolism	13	0.553
hsa00630:Glyoxylate and dicarboxylate metabolism	10	1.674
hsa01212:Fatty acid metabolism	13	3.191
hsa04930:Type II *diabetes mellitus*	13	3.191
hsa04973:Carbohydrate digestion and absorption	12	3.218
hsa00410:Beta-Alanine metabolism	10	3.666
hsa00270:Cysteine and methionine metabolism	11	3.888
hsa02010:ABC transporters	12	4.744

**Table 5:** Kegg pathways enriched in segment 4 versus 1 (FDR < 5%)

Term	Gene Count	FDR %
hsa04142:Lysosome	49	< 0.001
hsa04110:Cell cycle	31	0.004
hsa04721:Synaptic vesicle cycle	20	0.009
hsa00511:Other glycan degradation	9	0.2
hsa00670:One carbon pool by folate	9	0.5
hsa05110:Vibrio cholerae infection	15	0.7
hsa03460:Fanconi anemia pathway	15	0.7
hsa05120:Epithelial cell signaling in *Helicobacter pylori* infection	17	0.9
hsa03010:Ribosome	27	0.9
hsa04966:Collecting duct acid secretion	10	1.1
hsa03030:DNA replication	11	2.8

Similar results were observed following mapping against the KEGG database using the DAVID platform. Pathways related to general nutrient digestion like “Fat digestion and absorption” and “Citrate cycle (TCA cycle)” were among KEGG pathways enriched in segment 1. The ABC transporters were also enriched in segment 1. In contrast, lysosomal genes were highly enriched in segment 4 in addition to “one carbon pool by folate” and genes involved in responses to pathogens as indicated by “Vibrio cholera infection” and “Epithelial cell signaling in *Helicobacter pylori* infection”.

#### Expression of selected neuropeptides along the wrasse intestine

Most investigated neuropeptides were constitutively expressed along the length of the wrasse intestine (*pomc*, *npy, ccka, lep,*). Exceptions to this were both peptide yy (*pyy*) gene paralogs and motilin (LABE_00007846), which decreased in expression towards the posterior region, comparable to most of the genes involved in digestion and uptake of nutrients. Peptide Y a was among the top ten most highly enriched in the anterior part (segment 1) compared to segment 4 (Additional file 9). Also, cholecystokinin b (*cckb*) was mainly expressed in the most anterior intestinal segment (72 fold higher in segment 1 vs the other segments). In contrast, *ccka* was constitutively expressed along the intestine*.* The RNA-seq results found motilin mRNA expression was 6.7 fold higher in the anterior versus the posterior segment (segment 1 vs 4). The expression levels for the motilin receptor like gene, *ghsrb*, *pomc* and *lep* were too low for relative quantification.

#### Identification of vitamin B-12 binding proteins in wrasse

Genes involved in vitamin B12 (cobalamin) uptake cubulin (*cubn*)*,* amnion associated transmembrane protein (*amn*) and LDL receptor related protein 2 (*lrp2*), were among the top differentially expressed genes in wrasse intestine (Additional file 9). Other genes coding for key vitamin B12 uptake and transport proteins, including orthologues of the mammalian gastric intrinsic factor (GIF), transcobalamin 1 (TCN1) and 2 (TCN2) were also investigated. Orthologues for transcobalamin 2 (LABE_00042844) was identified, while *GIF* and *TCN1* were not, identified in the wrasse genome Table 6. However, a *tcn-like* gene (*tcnl*) (gene ID: Labe LABE_00037777) was identified in wrasse using BLAST searches in the same genomic region as the *tcnl* gene in other fish species. The *tcnl* gene was 67% similar between wrasse and *Larimichthys crocea* (e-value = 6E-45, BLASTX against the NCBI database). Wrasse *tcnl* was situated next to *abcg4* as found for *tcnl* in zebrafish (ENSDARG00000068088), platyfish (*Xiphophorus maculatus*) (ENSXMAG00000005938) and tetraodon (ENSTNIG00000011745). Lack of *tcn1* and *gif* has previously been shown in other teleosts [25]. The *tcnl* was constituently expressed through the intestine with no significant differences between the segments.

**Table 6:** Presence (+) and absence (−) of genes related to vitamin B-12 uptake in mammals, gastric teleosts and ballan wrasse

		Mammals	Gastric teleosts	Ballan Wrasse
GIF	gastric intrinsic factor	+	–	–
TCN1	Transcobalamin 1	+	–	–
TCN2	Transcobalamin 2	+	+	+
TCN-like	Transcobalamin like	–	+	+

## Discussion

### Loss of stomach coinciding with gene losses of related genes

We here present the successful generation of the ballan wrasse genome based on integration of long and short sequencing reads in addition to transcriptomic profiling of the wrasse intestine, revealing substantial gene losses regulating stomach function coinciding with the loss of an anatomical and functional stomach. Lost genes include those needed for acidic gastric environment such as the gastric proton pumps (*atp4*, *atp4b*) and pepsinogen-related genes, as previously reported lost in other agastric fish species and the duck-billed platypus (*Ornithorhynchus anatinus*) [26]. Furthermore, by comparing our wrasse data-sets to the genetic fingerprint for human stomach [20], we confirmed most genes related to stomach function; including pepsinogen A3, A4 and A5 (*PGA3, 4* and *5*)))*,* ATPase H+/K+ transporting alpha and beta subunits (*ATP4A* and *ATP4B*),)*GIF,* gastrin (*GAST*), lipase F gastric type (*LIPF*), trefoil factor 1 (*TFF1*) and progastrisin (*PGC*)); are lost in the wrasse genome*.* It should be noted that it is in general difficult to demonstrate the absence of a gene. There is always the chance that they remain undetected in regions difficult to sequence such as GS rich regions. GC-rich areas are hard to sequence, both with Illumina and Sanger approaches. However, PacBio sequencing is much better at covering these GC-rich regions. For instance in the case of bird genomes, of which the promotor region is often GC-rich. Older Sanger genome assemblies often had gaps in the GC-rich regions, but PacBio based assemblies cover these [21].

Mucin 5 AC, oligomeric mucus/gel-forming (*muc5ac*) was the only gene related to human stomach function found in wrasse. Furthermore, a gene like mammalian *MUC5AC*, neighboring mucin 2 (*muc2*), was identified in the wrasse genome assembly. The mammalian MUC5AC protein is a gel-forming mucin protein secreted in the gastrointestinal tract [27]. Orthologues of *muc5ac* were identified in most teleost species, including several agastric fish species (Ensembl database, release 86). However, our analysis failed to find *muc5ac* expression in wrasse intestine. Thus, *muc5ac* may be expressed at levels below the method detection limit in intestine of animals of the age/size used in the current study, or *muc5ac* may have another function in fish.

The loss of stomach accompanied with specific gene losses is not unique to labrids, and has been reported in a wide range of unrelated vertebrates [5, 26], indicative of convergent evolution. Gene losses, due to relaxation of purifying selection or genetic drift, is thought to be a major force driving evolution, facilitating phenotypic traits that adapt different species to their individual niches. Food source, specifically the calcareous diet of wrasse, is suggested as a possible driving force underlying stomach loss in this species [5]. However, overall the diets of agastric fish is essentially as diverse as for gastric species, and includes wood, detritus, invertebrates and fish [28–30]. Thus, if the loss of stomach function in wrasse was enabled due to their diet of easily digestible proteins and/or high inclusions of calcareous components, this cannot explain why stomach loss occurred in all agastric fish species. The large morphological variation of the intestine reported in agastric fish suggest stomach loss is a result of targeted selection, giving rise to new evolutionary roles of the intestine, enabling agastric teleost species to better occupy certain food niches than those with stomachs.

### Signaling pathways affecting appetite control in stomach-less fishes

The stomach is thought to be the major organ affecting appetite control, via the production of ghrelin, in both mammals and most fish species [31]. In mammals, ghrelin is referred to as the hunger hormone and works in concert with a range of other neuropeptides and hormones to control appetite. In the present study, we confirmed the lack of ghrelin in ballan wrasse and in other stomach-less fish species. The only exception is the cyprinids, where ghrelin is present [32–34]. In addition to ghrelin, the enzyme responsible for the acyl modification of activating ghrelin, MBOAT4, previously known as Ghrelin O-Acyl Transferase, is also missing from the wrasse genome assembly. MBOAT4 has been identified in several fish species [35], but not in the genomes of fish that have lost ghrelin. This shows that not only has ghrelin been lost, but also genes on different chromosomes associated with its function and maturation. Ghrelin is the only known orexigenic (stimulating food intake) hormone produced in the vertebrate digestive tract, and it is typically expressed in the stomach [32–34]. Both short-term and long-term fasting can increase ghrelin expression in fish stomach [31] and brain [36]. However, ghrelin is most highly expressed in pancreatic tissue in the zebrafish, an agastric cyprinid [34], which suggests ghrelin could play multiple roles in fish physiology.

Despite the absence of ghrelin in non-cyprinid agastric fish species), it is interesting to note that a gene coding for a ghrelin receptor was identified in ballan wrasse. The ballan wrasse ghrelin receptor occurred in the same conserved region as *ghrsb* in other teleost species including zebrafish. In zebrafish and mammals, the expression of the ghrelin receptor is elevated as a response to fasting. For example, in zebrafish, the ghrelin receptor expression levels increased in intestinal tissue but not in brain following 15 days of food deprivation [34]. No effect was observed after 17 h of fasting in the same study. In comparison, fasting and a negative energy balance raised *GHSR1* mRNA in rat brain tissue, while feeding restored it to its normal values [37]. In the present study, the abundance of *ghrsb* was very low compared to other intestinal G protein-coupled receptors such as the motilin receptor. Whether this is due to the fish being sampled in a fed state and with a positive energy balance, or to the lack of ghrelin, is not known. Which role the ghrelin receptor may play in the absence of its only confirmed ligand, ghrelin (reviewed by Kaiya et al. [38]), is also unknown. Cancer research has demonstrated alternative non-peptide ligands can bind to ghrelin receptors [39, 40], thus as yet unknown non-ghrelin compounds could potentially be ligands of ghrelin receptors in fish lacking ghrelin.

Motilin, a peptide hormone involved in the regulation of intestinal motility and peristalsis, was present in ballan wrasse. Motilin has previously been found in other agastric fish [24]. In mammals, motilin and acyl-ghrelin have overlapping functions. For example, along with intestinal motility, both hormones can stimulate growth hormone release in vitro and in vivo [41, 42], and accelerate gastric emptying [43, 44]. Despite some apparent overlapping functions, it is not known whether motilin plays an analogous role in regulating hunger in fish. In humans, it is suggested that motilin induce cyclic intestinal contractions during inter-digestive fasting act as a hunger signal [45] and thus orexigenic ghrelin function is overlapped by the motilin pathway. While both motilin and the motilin receptor were expressed along the whole length of the ballan wrasse intestine, it remains unclear if motilin plays a role in hunger regulation in fish as found in mammals.

In addition to ghrelin and motilin, several gut hormones and neuropeptides serve a dual role in regulating digestion and appetite control (see recent review by [46]). As found for motilin, *cckb*, *pyya* and *pyyb* were expressed throughout the wrasse intestine, with the highest expression in the foregut and the lowest in the hindgut. In mammals, PYY has an anorexic function by decreasing gut motility and appetite, and providing a satiety signal towards the end of a meal. Together with the glucagon-like peptide-1 (GLP-1), mammalian PYY is secreted by intestinal L-cells following food intake. Teleosts possess gene paralogs, *pyya* and *pyyb*, of the single mammalian PYY orthologue. In ballan wrasse, both *pyy* genes were highly expressed in the anterior intestine with a declining expression towards the posterior part, with only traces in the hind-gut segment. This contrasts with mammalian findings, where the mRNA expression of *PYY* increases towards the intestine posterior, with the highest expression in the colon [20, 47]. Interestingly, mammalian PYY and GLP-1 (glucagon-like peptide-1) are simultaneously expressed in the same L-type cells in the colon and rectum [47–49]. This co-localization of expression may be conserved from fishes to mammals, as the ballan wrasse intestinal expression pattern of *glp* follows that of the *pyy* paralogs. If so, why expression of the *pyy* and *glp* genes has shifted from the anterior intestine in fish to the colon in mammals remains unknown.

### Deciphering the digestive function of the wrasse intestine by transcriptome profiling

Agastric fish often have a bulbous structure in the intestine anterior which is referred to as the intestinal bulb in adult zebrafish [50]. This structure is thought to function primarily as a food store. Wrasse have an intestinal bulb, followed by an intestinal loop before the hind-gut (Additional 5). However, the first three segments of the wrasse intestine appear to act as one continuous short tube with little specialization in functional activity based on the expression patterns of digestion related genes found in our study. An expression gradient for digestion related genes between the segments was observed, with high expressions in the anterior intestine that declined in abundance towards the hind-gut. This pattern was especially clear for the apolipoprotein, phospholipase A2 and *pyya* genes among others. A similar pattern was observed in zebrafish using microarrays and qPCR [51]. Both KEGG analysis and gene ontology (GO) enrichment analysis found that the segments 1 to 3 are involved in multiple nutrient metabolism and uptake processes such as lipid metabolism, protein transport, cholesterol homeostasis, fat digestion and absorption. Furthermore, mechanisms involved in bile secretion were enriched in the intestine anterior.

The intestine of agastric cyprinids is usually divided into three functional segments. The anterior segment, which comprises up to 70% of the gut length, is where the main digestion and uptake of food occurs [52, 53]. The mid-segment (up to 25% of the total intestine length) is related to immune function. The main function of the final segment may be water and iron uptake [54]. An analogy to the zebrafish final segment was not found in ballan wrasse. However, the apparent function of the anterior segment in zebrafish correlates with the first three segments in ballan wrasse, and the hind-gut segment in wrasse corresponds to the mid-section of zebrafish. Unlike mammals, teleosts do not have a rudimentary colon. The colon, comprising the last part of the mammalian intestine, lacks digestive function and is thought to have evolved in terrestrial vertebrates primarily to conserve water [55]. However in fish, water is absorbed along the full length of the intestine through osmotic regulatory mechanisms involving bicarbonate transporters [56]. As with other teleosts, our data demonstrate that unlike the mammalian colon, the wrasse hind-gut has an apparent capacity for nutrient absorption. Interestingly, a colon-specific gene, membrane spanning 4-domains A12 (*ms4a12*) [20] was most highly expressed in the hind-gut of the wrasse. This is a cell-surface protein that is primarily found in the apical membrane of colonocytes and it may be an important factor in colonic development in mammals [57].

The acidic environment in the stomach is an important chemical barrier against ingested pathogens. However, there is no evidence that agastric teleosts are more susceptible to disease than their gastric counterparts. Our study found expression of immune-relevant genes; such as immunoglobulins, and immune-related receptors; along the full length of the wrasse intestine, with an increase of antigen-presenting capabilities in the hind-gut. Both KEGG and GO analysis of enriched genes in the hind-gut (segment 4) revealed increased endocytosis and lysosomal activity, which could be related to antigen processing and immune functions. One of the genes expressed most in the wrasse hind-gut (segment 4) compared to segments 1–3, was mannose receptor C-type 1 (*mrc1*). The *mrc1* protein mediates endocytosis of glycoproteins and pathogenic microbes by macrophages [58]. Unlike mammals which have well-defined gut-associated lymphoid tissue (GALT) structures such as Peyer’s patches, the immunological organization of GALT is more diffuse in teleosts [59]. However, most teleosts have defined areas which are recognized by enterocytes with high pinocytotic activity and supranuclear vacuoles capable of taking up large macromolecules. These defined areas are also highly infused with macrophages compared to other areas [60]. It was previously speculated that the uptake of macromolecules in cyprinids was connected to the loss of stomach and an inefficient protein digestion [53], but the uptake of macromolecules has been demonstrated in both agastric [60, 61] and gastric fish [52, 62, 63]. Based on *mrc1* being predominately expressed in segment 4, the GALT in wrasse may also be situated more posteriorly in the intestine than in other fish.

Transcriptome profiling revealed a high degree of DNA replication, indicative of increased cell division, in the hind-gut compared to the rest of the intestine. This suggests the highest rates of cell turnover occur in the posterior region of the intestine.

In addition to the lysosome and endocytosis-related genes, several key genes coding for proteins involved in the active uptake of vitamin B_12_ (cobalamin); Cubulin (*cubn*)*,* amnion associated transmembrane protein (*amn*), LDL receptor related protein 2 (*lrp2*); were among the top 10 most differentially expressed genes in the hind-gut. Another gene, *tcn2,* which is crucial for the efficient uptake and transport of cobalamin from the intestine [64], was also enriched in the hind-gut. In mammals, three cobalamin-binding proteins have been identified; TCN1 present in saliva and gastric fluids facilitates cobalamin transport through the stomach, GIF expressed in the stomach facilitates cobalamin transport through the middle intestine, and TCN2 facilitates cobalamin transport through the blood. On the other hand, no GIF orthologue has been identified in teleosts [65]. Although a haptocorrin gene has not been identified in fish, a *tcn-like* (*tcnl*) protein has been observed in both zebrafish [66] and rainbow trout (*Oncorhynchus mykiss*) [65]. Like gif and tcn1, the tcn-like protein in rainbow trout can protect cobalamin against low pH and is suggested to be a functional intermediate between gif and tcn1. The tcn-like protein is highly expressed in the stomach of rainbow trout. Interestingly, we identified the *tcn-like* gene in wrasse, and it was expressed consistently along the entire length of the intestine. In gastric animals, enzymatic cleaving of cobalamin from dietary proteins by gastric pepsin is crucial for its absorption [67]. Both studies on the side-effects of proton pump-inhibiting pharmaceuticals and the effects of gastric by-passes suggest that both low pH and pepsin are necessary for effective cobalamin uptake. How the uptake of cobalamin is handled in agastric animals lacking low pH, pepsin and *gif*, or even in gastric animals lacking just *gif*, remains unknown.

The ballan wrasse is currently being commercially cultured for its cleaner fish abilities in salmon production. However, cultured wrasse growth and development is suboptimal when fed commercial formulated diets developed for gastric fish species. Therefore, more basic knowledge on intestinal function of wrasse are needed. It has been hypothesized that the bulbous anterior part of the intestine has a stomach like function (pseuodogaster) mixing and storing food after a meal. There are no indications to such a function of the intestinal bulbous. On the contrary, this part of the intestine shows the highest expression of genes associated with digestion and absorption of nutrients, indicative of normal mid intestine functions. This could imply a need for continuous feeding as opposed to meals. This conclusion is supported by lack of ghrelin. In mammals, ghrelin is associated with meal based eating pattern, du to pre-prandial surges [68].

## Conclusions

Here we present the first genome sequenced of a labridae, a family of agastric fish representing more than 600 species. In addition, we generated a global transcriptional database of intestinal gene expression along the length of the ballan wrasse intestine. The study shows that the wrasse intestinal tract is functionally similar to the small intestine of mammals, and lacks both a stomach and a functional colon. With one exception (*muc5ac*), all genes linked to stomach function have been lost in the wrasse genome. Furthermore, we found *ghrelin* and *mboat4* both involved in digestion and appetite regulation have been lost in all agastric non-cyprinid teleosts. However, the multiple forms of neuropeptides identified in the present study indicate a complex appetite-regulating network in the gut-brain axis in fish, comparable to, if not exceeding the mammalian system in complexity. These findings increase our understanding of the evolution of the digestive tract, particularly in relation to appetite regulation. Aquaculture is expected to play a large role in supplying the increasing quantities of seafood needed by the global population. Findings from this study will aid the formulation of diets better suited to wrasse, which as cleaner fish will aid the sustainability of the global salmon industry.

## Methods

Gut segments of five adult ballan wrasse () (58 ± 5 g) and one brain sample from one adult ballan wrasse were sampled from a commercial fish farm (Marine Harvest Labrus,in Øygarden outside Bergen, Norway. The intestinal samples were used to generate de novo transcriptome assembly libraries and for transcriptomic profiling of the wrasse intestine. The brain sample was only used for the de novo transcriptome assembly. The fish were killed by a blow to the head, and the whole gut was removed and divided into four segments (Additional file 5). The fish had been fed Skretting Labrus feed (Skretting, Norway). All excised gut tissues were inspected for intestinal content to ensure digestive activity. Gut content was removed by rinsing each segment in PBS-buffered milli-Q water. For the transcriptomic characterization of the intestine, tissue samples from the four different intestine segments were excised. Each segment was flash-frozen in liquid nitrogen and stored at − 80 °C pending further processing.

### DNA library preparation and genome sequencing

One *Labrus bergylta* (30.7 g) from the Marine Harvest Labrus facility was killed by a blow to the head, and tissues were harvested and stored in liquid nitrogen until further processing. DNA was extracted from muscle tissue using the DNeasy blood and tissue kit (Qiagen Inc., Valencia, CA). The quality (260/280: 1.91–1.96 and 260/230 < 1.96) and amount of DNA were analyzed using a NanoDrop® ND-1000 spectrophotometer (NanoDrop Technologies, Wilmington, DE, USA), a Qubit fluorometer and the dsDNA High Sensitivity Assay Kit (Life Technologies Corp. Carlsbad, CA, USA) respectively. The DNA integrity was verified on an 0.8% agarose gel over night.

#### Sequencing

A combination of two different platforms; the Illumina HighSeq2500 and the PacBio RS II platform; were used for the genome sequencing.

For Illumina library preparation, ≈2 μg genomic DNA was fragmented on a Covaris AFA E220 system (Covaris Inc., MA, USA) down to an average fragment length of ≈550 bp. The fragmented sample was converted into a library using a Kapa Hyper PCR-free library preparation protocol, including the dual SPRI size selection step (Kapa Biosystems, MA, USA). The final library was cleaned twice (0.7:1, bead: DNA ratio) with Agencourt AMPure XP (Beckman Coulter Inc., CA, USA), before library QC on an Agilent 2100 Bioanalyser (Agilent Technologies, CA, USA), and quantification using the Kapa Library quantification Kit (Kapa Biosystems, MA, USA). Clustering and sequencing were performed on one flowcell on a Hiseq 2500 in Rapid Mode (250 bp PE) using on-board clustering according to protocols (Illumina Inc., CA, USA). Approximately 90 Gbp of data was produced.

For library preparation for the PacBio RS II platform, 5 μg of the extracted DNA was sheared into 20 kb fragments using HydroShear (Genemachines, Thermo Fisher Scientific Inc., MA, USA). SMRTbell template library was prepared using the Pacific Biosciences 20 kb library preparation protocol. The library was size selected for a lower cutoff of 7 kb using BluePippin (Sage Science, MA, USA). Sequencing was performed on the PacBio RS II (Pacific Biosciences of California Inc., Menlo Park, CA, USA) using P6 polymerase binding and C4 sequencing kits with a 360 min acquisition. In total, 15 SMRT cells were sequenced, and ≈26.8 Gbp of library bases were produced.

#### Assembly

PacBio reads were assembled using a snapshot of canu (https://github.com/marbl/canu/) [15] from December 30th 2015. The genomeSize option was set to 700 Mbp, while all other options were default.

The 90 Gbp of Illumina sequencing data were mapped to scaffolds, and Pilon [17] was used to close gaps and recall consensus. Because of the high coverage in Illumina reads (~ 120×), only half (one lane) were used in scaffolding by the SGA scaffold module [16]. The reads were mapped to the canu assembly using BWA-MEM 0.7.12 [69] with the –M option to mark short split hits as secondary, and scaffolding was performed with default options. Illumina reads were then mapped to the scaffolds using BWA-MEM 0.7.12 [69], and Pilon version 1.16 [17] was applied to improve consensus sequence and close/reduce gaps.

#### Validation of the genome assembly

Two tools; CEGMA version 2.4.010312 [70, 71] with default options, and BUSCO v1.1b [72] using an Actinopterygii specific gene set; were applied to the genome assembly to assess its quality in terms of the number of conserved eukaryotic genes recovered.

#### Repeat library

A library of repeats was created as described in Tørresen et al. [73]. Briefly, RepeatModeler open-1.0 (http://www.repeatmasker.org), LTRharvest [74], and TransposonPSI (http://transposonpsi.sourceforge.net), were used to create a set of putative repetitive elements. Repetitive elements that matched against an UniProtKB/SwissProt database and not against the repeat peptide database included in RepeatMasker (http://www.repeatmasker.org) were removed, and the remaining sequences were classified and combined with known eukaryotic repeat sequences from RepBase (version 20,150,807) [75], before being used in gene annotation. The genome was then submitted to the European Nucleotide Archive (accession number: PRJEB13687).

#### Genome annotation

A two-pass iteration with MAKER version 2.31.8 [18, 19] was performed on the final genome assembly as described at https://github.com/sujaikumar/assemblage/blob/master/README-annotation.md, and in Campell et al. [76], with some minor modifications. First, three ab initio gene finders were trained. SNAP version 20,131,129 [77] was trained on the genes found by CEGMA version 2.4.010312 [70, 71]. GeneMark-ES version 2.3e [78] was trained on the genome assembly itself. And AUGUSTUS version 3.0.2 [79, 80] was trained with the Actinopterygii gene set from BUSCO v1.1b [72] by using the –long option for BUSCO. SwissProt/UniProtKB [81] was downloaded on the 27th of January 2016 (release 2016_01), and used as an input to MAKER, together with two sets of transcriptomes (one assembled with Trinity and the other based on 454 reads, as described below), the repeat library, and the trained gene finders, for the first pass of training. SNAP and AUGUSTUS were then retrained on the GFF output of MAKER, and a second pass was performed. Gene ontologies, gene families and domains were applied to the protein output of the second pass by running InterProScan version 5.4–47 [82], and putative gene names were identified by BLAST against SwissProt/UniProtKB. Gene models with AED of more than 0.5 were removed, resulting in a final set of 31,050 gene models. Annotation files are available at https://search.datacite.org/works/10.6084/M9.FIGSHARE.5032226.V1 (DOI: 10.6084/m9.figshare.5032226).

### RNA extraction, RNA sequencing and de novo transcriptome assembly

#### RNA extraction

The intestinal tissues and brain were homogenized using zirconium beads (CK28, 4 mm) in a Precellys 24 homogenizer (Bertin Technologies, Montigny-le-Bretonneux, France). Total RNA was then extracted from the homogenates using a BioRobot® EZ1 and RNA Tissue Mini Kit (Qiagen, Hilden, Germany). The samples were then DNase treated according to the manufacturer’s instructions and eluted in 50 μL RNase-free water. RNA quality was assessed using a NanoDrop ND-1000 UV–vis Spectrophotometer (NanoDrop Technologies, Wilmington, DE, USA). The average 260/280 and 260/230 nm ratios for the total RNA samples were 2.19 ± 0.12 and 2.1 ± 0.07 (Mean ± STD), respectively. RNA integrity was analyzed using an Agilent 2100 Bioanalyzer and RNA 6000 Nano LabChip kit (Agilent Technologies, Palo Alto, CA, USA). The RNA integrity number (RIN) was 7.8 ± 0.1 (mean ± STD) for the 24 evaluated samples. The RNA was then stored at − 80 °C.

#### Illumina RNA seq library preparation and sequencing

Library preparation and sequencing were performed by the Norwegian Sequencing Centre, Oslo, Norway (www.sequencing.uio.no). Libraries were prepared using TruSeq RNA v2 reagents (Illumina) according to the manufacturer’s instructions. Libraries were sequenced on an Illumina HiSeq 2000 using V3 clustering and sequencing reagents according to the manufacturer’s instructions. Image analysis and base calling were both performed using Illumina’s RTA software version 1.17.21.3. Reads were filtered to remove those with low base call quality using Illumina’s default chastity criteria.

#### De novo Illumina transcriptome assembly and mapping

Sequence adaptors were removed using Cutadapt [83] and default parameters (Cutadapt commands are presented in supplementary 1). Bad reads and sequence regions were filtered and trimmed using Sickle (https://github.com/najoshi/sickle) with a 40 bp minimum remaining sequence length, Sanger quality of 20, and no 5′ end trimming. Prior to assembly, read quality was checked using fastQC version 0.9.2 (The FastQC project, http://www.bioinformatics.babraham.ac.uk/projects/fastqc/) to ensure improvement compared to the raw data. De novo transcriptome assemblies of a total 252 M trimmed and filtered paired end sequence reads derived from 5 intestinal libraries and 7 brain libraries were conducted using the Trinity pipeline [84] with default parameters(). The transcriptomic assemblies were additionally filtered for small and redundant sequences by first aligning and estimating abundance for each transcript (align_and_estimate_abundance.script) and then filtering using the “filter_fasta_by_rsem_values.pl” trinity script. This Transcriptome Shotgun Assembly project has been deposited at DDBJ/EMBL/GenBank under the accession GFFM00000000. The version described in this paper is the first version, GFFM01000000.

#### De novo 454 transcriptome assembly

For genome annotation, a 454 transcriptome was included in this study. A 454 GS FLX transcriptome was generated by a commercial supplier (IGSP Genome Sequencing and Analysis Core Facility, Duke University, USA) using RNA isolates from whole juvenile ballan wrasse. Normalized cDNA libraries and sequencing were conducted as previously described [85]. The reads were assembled and analyzed using the Newbler software [86]. This Transcriptome Shotgun Assembly project has been deposited at DDBJ/EMBL/GenBank under the accession GFDP00000000. The version described in this paper is the first version, GFDP01000000.

#### RNA seq mapping and abundance estimates

Each gut segment RNAseq library (36) was mapped individually to the ballan wrasse genome assembly (European Nucleotide Archive accession number: PRJEB13687, http://www.ebi.ac.uk/ena/data/view/PRJEB13687) using the Tophat2 short read aligner [87]. Transcript abundances were estimated using the FeatureCounts [88] software of the Subread package (http://subread.sourceforge.net/). An average of 67 ± 3% of the reads in each sample were mapped to the de novo genome assembly by Tophat and counted by FeatureCounts.

#### Search for the presence of genes related to stomach function

Searching for genes known to be involved in stomach function was conducted using a combination of BLAST search and looking at synteny. A outline of the search strategy can be found in Additional file 3. Candidate genes (PGA3, PGA4, PGA5, ATP4A,ATP4B, GIF, GAST, LIPF, TFF1, PGC, MUC5AC) were selected based on previous studies from both fish and mammals identifying stomach related gene expression [5, 19]. We also aimed to identify genes known to be involved in Vitamin B12 uptake (GIF, TCN1 and TCN2) and appetite regulation (GHRL, GHRS, MBOAT4, MNL, MLNR, PYY, CCK, NPY, POMC, LEP).

#### Differential expression analysis and statistics

Read counts of the intestinal segment samples were further processed and analyzed using the Bioconductor - DESeq2 analysis package [89]. Genes with read counts < 1 in all samples were excluded from further analysis prior to normalization and differential expression (DE) analysis leaving a total of 27,392 transcripts for further analysis. Differences in expression between the intestinal segments were analyzed using the DESeq2 package, and genes with adjusted *p* values (p-adjust < 0.05) were further analysis using DAVID Bioinformatics Resources 6.8 (Beta) with default settings (GO and KEGG pathway analysis). The 1000 most significantly differentially expressed genes (adjusted p value (q-value) < 1.7e-4) were visualized using hierarchical clustering implemented in the Qlucore Omics explorer software package version 3.2 (Qlucore AB, Lund, Sweden). Graph was constructed using GraphPad Prism (V.7., Graphpad Software. San Diego, USA).

## Additional files

Additional file 1: Illumina de novo brain and intestine transcriptome assembly statistics. (PDF 154 kb)

Additional file 2: Data showing full length analysis of the Illumina de novo brain and intestine transcriptome assembly using BLAST+ and Uniprot as a reference database (release). The metric analysis examines the number of assembled transcripts that appeared to be full length or nearly full length. The first column (**#hit_pct_cov_bin %)** shows the present coverage in the database. The second column (**count_in_bin)** shows the number proteins that match a trinity assembly transcript by more than the present given in the table >%. E.g 1554 proteins match a transcript by > 80% and <= 90% of their protein lengths. The third column (>bin_below) shows the number of protein in the reference database that are represented by nearly full length transcripts with a % alignment coverage. (PDF 285 kb)

Additional file 3: Schematic figure showing the strategy for gene search and for deciding whether a gene is present (“Present”) in the wrasse genome or not (“Not present”). (PNG 322 kb)

Additional file 4: The figures shows the genomic region with Ensembl coordinates and flanking genes (Synteny) for *mboat4*, motilin receptor (*mlnr*), peptide y a (*pyya*), peptide y b (*pyyb*), cholecystokinin a (*ccka*), cholecystokinin b (*cckb*). (PDF 333 kb)

Additional file 5: Figure showing the digestive organs of the Ballan wrasse and a schematic depiction of the collected intestinal segments. (TIFF 3090 kb)

Additional file 6: The table shows the differentially expressed genes between the different segments. Segment 2 versus (vs) 1, 3 vs 1, 4 vs 1, 3 vs 4 and 3 vs 2. Gene IDs, gene symbol, *P*-values, P-adjust values and Log2 fold change are given in the table. Expression values are given in the sheet labeled “transcriptome” (XLSX 5003 kb)

Additional file 7: Excel file showing the statistics of the functional enrichment analysis using the DAVID tool. Number of genes counted for each term (count) P-values, list of genes counted (Genes), p adjust values (Benjamini) and % false discovery rate (FDR) are given in the tables. The figure shows enriched gene ontology terms (GOs) (biological function (BF), cellular compartment (CC) and molecular function (MF)) and KEGG pathways in segment 4 (seg4 vs seg1), segment 1 (seg1 vs 4) and segment 3 vs segment 2. It also shows the complete list of terms used for the Revigo analysis. (XLSX 112 kb)

Additional file 8: Clustering of gene ontology terms (GOs) enriched in segment 1 (Fig. A) and segment 4 (Fig. B). REVIGO summarizing and visualizing software [90] was used to construct a tree map clustering of gene ontology terms (GO) enriched in the hind gut (segment 1) of ballan wrasse intestine using. Box size indicate the relative size of the GO cluster. Colors are added to separate the main hierarchy GO terms. The figure indicates the largest enriched clusters of GO terms in segment 1 and 4. (PDF 443 kb)

Additional file 9: Top ten differentially expressed genes based on fold change (positive or negative) between segment 1 and 4 (Upregulated and downregulated genes in segment 1 compared to 4. The table shows Gene_ID, gene_symbol, Fold change and p-adjust values. (PDF 258 kb)

## Abbreviations

*abcg4*: Atp binding cassette subfamily g member 4
*amn*: Amnion associated transmembrane protein
Bp: Base pairs
cck: Cholecystokinin
*ccka*: Cholecystokinin a
*cckb*: Cholecystokinin b
*cubn*: Cubulin
*FDR*: False discovery rate
ghrl: Ghrelin ligand
ghsr: Ghrelin receptor
*ghsrb*: Ghrelin receptor b
*gif*: Gastric intrinsic factor
*GO*: Gene ontology
Kbp: Kilo base pairs
lep: Leptin
*Lrp2*: Ldl receptor related protein 2
mboat4: Membrane bound o
Mbp: Mega base pairs
mln: Motilin
mlnr: Motilin receptor
*mrc1*: Mannose receptor c-type 1
*ms4a12*: Membrane spanning 4-domains a12
*muc2*: Mucin 2, oligomeric mucus/gel-forming
*muc5ac*: Mucin 5 ac, oligomeric mucus/gel-forming
npy: Neuropeptide y
*pga3*: Pepsinogen a3
*pga4*: Pepsinogen a4
*pga5*: Pepsinogen a5
pomc: Proopiomelanocortin
*pyy*: Peptide yy
*slc15A1*: Solute carrier family 15 member 1
*slc15A2*: Solute carrier family 15 member 2
*tcn1*: Transcobalamin 1
*tcn2*: Transcobalamin 2
*tcnl*: Transcobalamin-like
*tmprss15*: Transmembrane protease serine 15

## References

[1] Parenti P, Randall JE (2000). Bioline international official site (site up-dated regularly). Ichthyol Bull.

[2] Artüz ML: Age and growth of the ballan wrasse *Labrus bergylta Ascanius* 1767. 2007:1–5.

[3] Bjordal A: Sea lice infestation on farmed salmon: possible use of cleaner-fish as an alternative method for de-lousing. Canadian technical report of fisheries and aquatic … 1990.

[4] Treasurer JW (2002). A review of potential pathogens of sea lice and the application of cleaner fish in biological control. Pest Manag Sci.

[5] Castro LF, Goncalves O, Mazan S, Tay BH, Venkatesh B, Wilson JM (2014). Recurrent gene loss correlates with the evolution of stomach phenotypes in gnathostome history. Proc Biol Sci.

[6] Wilson JM, Castro LFC (2010). Morphological diversity of the gastrointestinal tract in fishes. Fish Physiol.

[7] Koelz HR (1992). Gastric-acid in vertebrates. Scand J Gastroentero.

[8] Soybel DI (2005). Anatomy and physiology of the stomach. Surg Clin N Am.

[9] O'Connor A, O'Morain C (2014). Digestive function of the stomach. Dig Dis.

[10] Ao J, Mu Y, Xiang L-X, Fan D, Feng M, Zhang S, Shi Q, Zhu L-Y, Li T, Ding Y (2015). Genome sequencing of the perciform fish *Larimichthys crocea* provides insights into molecular and genetic mechanisms of stress adaptation. PLoS Genet.

[11] Rondeau EB, Minkley DR, Leong JS, Messmer AM, Jantzen JR, von Schalburg KR, Lemon C, Bird NH, Koop BF (2014). The genome and linkage map of the northern pike (*Esox lucius*): conserved synteny revealed between the salmonid sister group and the neoteleostei. PLoS One.

[12] Tørresen OK, Star B, Jentoft S, Reinar WB, Grove H, Miller JR, Walenz BP, Knight J, Ekholm JM, Peluso P (2017). An improved genome assembly uncovers prolific tandem repeats in Atlantic cod. BMC Genomics.

[13] (NFR) NRC: FUGE II – Functional genomics in norway. Action plan 2007–2011**.** In*.*; 2007.

[14] Vij S, Kuhl H, Kuznetsova IS, Komissarov A, Yurchenko AA, Van Heusden P, Singh S, Thevasagayam NM, Prakki SRS, Purushothaman K (2016). Chromosomal-level assembly of the asian seabass genome using long sequence reads and multi-layered scaffolding. PLoS Genet.

[15] Koren S, Walenz BP, Berlin K, Miller JR, Phillippy AM: Canu: scalable and accurate long-read assembly via adaptive k-mer weighting and repeat separation. bioRxiv 2016. 10.1101/gr.215087.116 PMC5411767 28298431

[16] Simpson JT, Durbin R (2012). Efficient *de novo* assembly of large genomes using compressed data structures. Genome Res.

[17] Walker BJ, Abeel T, Shea T, Priest M, Abouelliel A, Sakthikumar S, Cuomo CA, Zeng Q, Wortman J, Young SK (2014). Pilon: an integrated tool for comprehensive microbial variant detection and genome assembly improvement. PLoS One.

[18] Holt C, Yandell M (2011). MAKER2: an annotation pipeline and genome-database management tool for second-generation genome projects. BMC Bioinformatics.

[19] Campbell MS, Law MY, Holt C, Stein JC, Moghe GD, Hufnagel DE, Lei JK, Achawanantakun R, Jiao D, Lawrence CJ (2014). MAKER-P: a tool kit for the rapid creation, management, and quality control of plant genome annotations. Plant Physiol.

[20] Gremel G, Wanders A, Cedernaes J, Fagerberg L, Hallstrom B, Edlund K, Sjostedt E, Uhlen M, Ponten F (2015). The human gastrointestinal tract-specific transcriptome and proteome as defined by RNA sequencing and antibody-based profiling. J Gastroenterol.

[21] Korlach J, Gedman G, Kingan SB, Chin CS, Howard JT, Audet JN, Cantin L, Jarvis ED (2017). De novo PacBio long-read and phased avian genome assemblies correct and add to reference genes generated with intermediate and short reads. Gigascience.

[22] Arcamone N, Neglia S, Gargiulo G, Esposito V, Varricchio E, Battaglini P, De Girolamo P, Russo F (2009). Distribution of ghrelin peptide in the gastrointestinal tract of stomachless and stomach-containing teleosts. Microsc Res Tech.

[23] Miura T, Maruyama K, Kaiya H, Miyazato M, Kangawa K, Uchiyama M, Shioda S, Matsuda K (2009). Purification and properties of ghrelin from the intestine of the goldfish, *Carassius auratus*. Peptides.

[24] Liu Y, Li S, Huang X, Lu D, Liu X, Ko W-h, Zhang Y, Cheng CHK, Lin H (2013). Identification and characterization of a motilin-like peptide and its receptor in teleost. Gen Comp Endocr.

[25] Lopes-Marques M, Ruivo R, Delgado I, Wilson JM, Aluru N, Castro LF (2015). Basal Gnathostomes provide unique insights into the evolution of vitamin B12 binders. Genome Biol Evol.

[26] Ordonez GR, Hillier LW, Warren WC, Grutzner F, Lopez-Otin C, Puente XS (2008). Loss of genes implicated in gastric function during *platypus* evolution. Genome Biol.

[27] Sinha J, Cao Z, Dai J, Tang H, Partyka K, Hostetter G, Simeone DM, Feng Z, Allen PJ, Brand RE (2016). A gastric glycoform of MUC5AC is a biomarker of mucinous cysts of the pancreas. PLoS One.

[28] German DP, Bittong RA (2009). Digestive enzyme activities and gastrointestinal fermentation in wood-eating catfishes. J Comp Physiol B.

[29] Horn MH, Gawlicka AK, German DP, Logothetis EA, Cavanagh JW, Boyle KS (2006). Structure and function of the stomachless digestive system in three related species of new world silverside fishes (Atherinopsidae) representing herbivory, omnivory, and carnivory. Mar Biol.

[30] Day R, German D, Manjakasy J, Farr I, Hansen M, Tibbetts I (2011). Enzymatic digestion in stomachless fishes: how a simple gut accommodates both herbivory and carnivory. J Comp Physiol B.

[31] Terova G, Rimoldi S, Bernardini G, Gornati R, Saroglia M (2008). Sea bass ghrelin: molecular cloning and mRNA quantification during fasting and refeeding. Gen Comp Endocr.

[32] Kojima M, Hosoda H, Date Y, Nakazato M, Matsuo H, Kangawa K (1999). Ghrelin is a growth-hormone-releasing acylated peptide from stomach. Nature.

[33] Cowley MA, Smith RG, Diano S, Tschop M, Pronchuk N, Grove KL, Strasburger CJ, Bidlingmaier M, Esterman M, Heiman ML (2003). The distribution and mechanism of action of ghrelin in the CNS demonstrates a novel hypothalamic circuit regulating energy homeostasis. Neuron.

[34] Eom J, Hong M, Cone RD, Song Y (2013). Zebrafish ghrelin is expressed in pancreatic endocrine cells and regulated by metabolic state. Biochem Bioph Res Co.

[35] Hatef A, Yufa R, Unniappan S (2015). Ghrelin o-acyl transferase in zebrafish is an evolutionarily conserved peptide upregulated during calorie restriction. Zebrafish.

[36] Amole N, Unniappan S (2009). Fasting induces preproghrelin mRNA expression in the brain and gut of zebrafish, *Danio rerio*. Gen Comp Endocr.

[37] Kim MS, Yoon CY, Park KH, Shin CS, Park KS, Kim SY, Cho BY, Lee HK (2003). Changes in ghrelin and ghrelin receptor expression according to feeding status. Neuroreport.

[38] Kaiya H, Kangawa K, Miyazato M (2013). Ghrelin receptors in non-mammalian vertebrates. Front Endocrinol (Lausanne).

[39] Su J, Geng J, Bao J, Tang Y, Liu M, Yu H, Han Y, Huang W, Zhou S (2016). Two ghrelin receptor agonists for adults with malnutrition: a systematic review and meta-analysis. Nutr J.

[40] Sanger GJ, Furness JB (2016). Ghrelin and motilin receptors as drug targets for gastrointestinal disorders. Nat Rev Gastroenterol Hepatol.

[41] Samson WK, Lumpkin MD, Nilaver G, Mccann SM (1984). Motilin - a novel growth-hormone releasing agent. Brain Res Bull.

[42] Samson WK, Lumpkin MD, McCann SM (1982). Motilin stimulates growth hormone release in vitro. Brain Res Bull.

[43] Christofides ND, Modlin IM, Fitzpatrick ML, Bloom SR (1979). Effect of motilin on the rate of gastric emptying and gut hormone release during breakfast. Gastroenterology.

[44] Christofides ND, Long RG, Fitzpatrick ML, McGregor GP, Bloom SR (1981). Effect of motilin on the gastric emptying of glucose and fat in humans. Gastroenterology.

[45] Tack J, Deloose E, Ang D, Scarpellini E, Vanuytsel T, Van Oudenhove L, Depoortere I (2016). Motilin-induced gastric contractions signal hunger in man. Gut.

[46] Volkoff H. The neuroendocrine regulation of food intake in fish: a review of current knowledge. Front Neurosci-Switz. 2016;10 10.3389/fnins.2016.00540 PMC5126056 27965528

[47] Böttcher G, Sjölund K, Ekblad E, Håkanson R, Schwartz TW, Sundler F (1984). Coexistence of peptide YY and glicentin immunoreactivity in endocrine cells of the gut. Regul Peptides.

[48] Bottcher G, Alumets J, Hakanson R, Sundler F (1986). Co-existence of glicentin and peptide YY in colorectal L-cells in cat and man. An electron microscopic study. Regul Pept.

[49] Rozengurt N, Wu SV, Chen MC, Huang C, Sternini C, Rozengurt E (2006). Colocalization of the α-subunit of gustducin with PYY and GLP-1 in L cells of human colon. Am J Physiol-Gastr L.

[50] Wallace KN, Akhter S, Smith EM, Lorent K, Pack M (2005). Intestinal growth and differentiation in zebrafish. Mech Dev.

[51] Wang Z, Du J, Lam SH, Mathavan S, Matsudaira P, Gong Z (2010). Morphological and molecular evidence for functional organization along the rostrocaudal axis of the adult zebrafish intestine. BMC Genomics.

[52] Stroband HW, Kroon AG (1981). The development of the stomach in *clarias lazera* and the intestinal absorption of protein macromolecules. Cell Tissue Res.

[53] HWJ S, Hvd M, Timmermans LPM (1979). Regional functional differentiation in the gut of the grasscarp, *Ctenopharyngodon idella* (Val.). Histochemistry.

[54] Stroband HW, Debets FM (1978). The ultrastructure and renewal of the intestinal epithelium of the juvenile grasscarp, *Ctenopharyngodon idella* (Val.). Cell Tissue Res.

[55] Grosell M, Farrell AP, Brauner CJ: Fish physiology: the multifunctional gut of fish; 2010.

[56] Kurita Y, Nakada T, Kato A, Doi H, Mistry AC, Chang M-H, Romero MF, Hirose S (2008). Identification of intestinal bicarbonate transporters involved in formation of carbonate precipitates to stimulate water absorption in marine teleost fish. Am J Physiol - Reg I.

[57] Koslowski M, Tureci O, Huber C, Sahin U. Selective activation of tumor growth-promoting Ca2+ channel MS4A12 in colon cancer by caudal type homeobox transcription factor CDX2. Mol Cancer. 2009;8 10.1186/1476-4598-8-77 PMC2759907 19781065

[58] Fraser IP, Koziel H, Ezekowitz RAB (1998). The serum mannose-binding protein and the macrophage mannose receptor are pattern recognition molecules that link innate and adaptive immunity. Semin Immunol.

[59] Rombout JHWM, Abelli L, Picchietti S, Scapigliati G, Kiron V (2011). Teleost intestinal immunology. Fish Shellfish Immun.

[60] Rombout JH, Lamers CH, Helfrich MH, Dekker A, Taverne-Thiele JJ (1985). Uptake and transport of intact macromolecules in the intestinal epithelium of carp (*Cyprinus carpio* L.) and the possible immunological implications. Cell Tissue Res.

[61] Rombout JH, van den Berg AA (1985). Uptake and transport of ferritin in the epithelium of carp (*Cyprinus carpio* L.) and the possible immunological implications. Cell Biol Int Rep.

[62] Georgopoulou U, Dabrowski K, Sire MF, Vernier JM (1988). Absorption of intact proteins by the intestinal epithelium of trout, *Salmo gairdneri*. A luminescence enzyme immunoassay and cytochemical study. Cell Tissue Res.

[63] Noaillac-Depeyre J, Gas N (1976). Electron microscopic study on gut epithelium of the tench (*Tinca tinca* L.) with respect to its absorptive functions. Tissue Cell.

[64] Nielsen MJ, Rasmussen MR, Andersen CBF, Nexo E, Moestrup SK (2012). Vitamin B-12 transport from food to the body's cells-a sophisticated, multistep pathway. Nat Rev Gastro Hepat.

[65] Greibe E, Fedosov S, Sorensen BS, Hojrup P, Poulsen SS, Nexo E (2012). A single rainbow trout cobalamin-binding protein stands in for three human binders. J Biol Chem.

[66] Greibe E, Fedosov S, Nexo E. The cobalamin-binding protein in zebrafish is an intermediate between the three cobalamin-binding proteins in human. PLoS One. 2012;7(4) 10.1371/journal.pone.0035660 PMC3331988 22532867

[67] Heidelbaugh JJ (2013). Proton pump inhibitors and risk of vitamin and mineral deficiency: evidence and clinical implications. Therapeutic Advances in Drug Safety.

[68] Cummings DE, Weigle DS, Frayo RS, Breen PA, Ma MK, Dellinger EP, Purnell JQ (2002). Plasma ghrelin levels after diet-induced weight loss or gastric bypass surgery. N Engl J Med.

[69] Li H. Aligning sequence reads, clone sequences and assembly contigs with BWA-MEM. arXiv:1303.3997v1 [q-bio.GN] (https://github.com/lh3/bwa). 2013.

[70] Parra G, Bradnam K, Ning Z, Keane T, Korf I (2008). Assessing the gene space in draft genomes. Nucleic Acids Res.

[71] Parra G, Bradnam K, Korf I (2007). CEGMA: a pipeline to accurately annotate core genes in eukaryotic genomes. Bioinformatics.

[72] Simão FA, Waterhouse RM, Ioannidis P, Kriventseva EV, Zdobnov EM. BUSCO: assessing genome assembly and annotation completeness with single-copy orthologs. Bioinformatics. 2015;31(19):3210-12. 10.1093/bioinformatics/btv351 26059717

[73] Torresen OK, Star B, Jentoft S, Reinar WB, Grove H, Miller JR, Walenz BP, Knight J, Ekholm JM, Peluso P, et al. An improved genome assembly uncovers prolific tandem repeats in Atlantic cod. BMC Genomics. 2017;18 10.1186/s12864-016-3448-x PMC5241972 28100185

[74] Ellinghaus D, Kurtz S, Willhoeft U (2008). LTRharvest, an efficient and flexible software for de novo detection of LTR retrotransposons. BMC Bioinformatics.

[75] Jurka J, Kapitonov VV, Pavlicek A, Klonowski P, Kohany O, Walichiewicz J (2005). Repbase update, a database of eukaryotic repetitive elements. Cytogenet Genome Res.

[76] Campbell MS, Holt C, Moore B, Yandell M: Genome annotation and curation using MAKER and MAKER-P. In: *Current Protocols in Bioinformatics.* John Wiley & Sons, Inc.; 2002. 10.1002/0471250953.bi0411s48 PMC4286374 25501943

[77] Korf I (2004). Gene finding in novel genomes. BMC Bioinformatics.

[78] Lomsadze A, Ter-Hovhannisyan V, Chernoff YO, Borodovsky M (2005). Gene identification in novel eukaryotic genomes by self-training algorithm. Nucleic Acids Res.

[79] Stanke M, Waack S: Gene prediction with a hidden Markov model and a new intron submodel. Bioinformatics 2003, 19(suppl 2):ii215-ii225. 10.1093/bioinformatics/btg1080 14534192

[80] Stanke M, Diekhans M, Baertsch R, Haussler D (2008). Using native and syntenically mapped cDNA alignments to improve de novo gene finding. Bioinformatics.

[81] UniProt: a hub for protein information. Nucleic Acids Res 2014, 43(D1):D204-D212. 10.1093/nar/gku989 PMC4384041 25348405

[82] Jones P, Binns D, Chang H-Y, Fraser M, Li W, McAnulla C, McWilliam H, Maslen J, Mitchell A, Nuka G (2014). InterProScan 5: genome-scale protein function classification. Bioinformatics.

[83] Martin M: Cutadapt removes adapter sequences from high-throughput sequencing reads, vol. 17; 2011.

[84] Grabherr MG, Haas BJ, Yassour M, Levin JZ, Thompson DA, Amit I, Adiconis X, Fan L, Raychowdhury R, Zeng Q (2011). Full-length transcriptome assembly from RNA-Seq data without a reference genome. Nat Biotech.

[85] Künstner A, Wolf JBW, BackstrÖM N, Whitney O, Balakrishnan CN, Day L, Edwards SV, Janes DE, Schlinger BA, Wilson RK (2010). Comparative genomics based on massive parallel transcriptome sequencing reveals patterns of substitution and selection across 10 bird species. Mol Ecol.

[86] Margulies M, Egholm M, Altman WE, Attiya S, Bader JS, Bemben LA, Berka J, Braverman MS, Chen YJ, Chen ZT (2005). Genome sequencing in microfabricated high-density picolitre reactors. Nature.

[87] Langmead B, Trapnell C, Pop M, Salzberg SL (2009). Ultrafast and memory-efficient alignment of short DNA sequences to the human genome. Genome Biol.

[88] Liao Y, Smyth GK, Shi W (2014). FeatureCounts: an efficient general purpose program for assigning sequence reads to genomic features. Bioinformatics.

[89] Love MI, Huber W, Anders S (2014). Moderated estimation of fold change and dispersion for RNA-seq data with DESeq2. Genome Biol.

[90] Supek F, Bosnjak M, Skunca N, Smuc T (2011). REVIGO summarizes and visualizes long lists of gene ontology terms. PLoS One.

