# Supplementary material for: Loss of stomach, loss of appetite? Sequencing of the ballan wrasse (Labrus bergylta) genome and intestinal transcriptomic profiling illuminate the evolution of loss of stomach function in fish
Source: BMC Genomics. 2018 Mar 6;19:186. doi: 10.1186/s12864-018-4570-8 (PMC5840709; doi:10.1186/s12864-018-4570-8)
Supplement: Supplementary file 1 — Illumina de novo brain and intestine transcriptome assembly statistics. (PDF 154 kb) [file 12864_2018_4570_MOESM1_ESM.pdf]

Illumina de novo brain and intestine transcriptome assembly statistics

| | Before filtering ((bp)) | After filtering | RSEM |
|---|---|---|---|
| Trinity genes | 174273 | 41235 | |
| Trinity transcripts | 262584 | 60007 | |
| N50 length | 2699 | 2794 | |
| Avearage contig length | 699.77 | 1699 | |
