# Supplementary material for: Loss of stomach, loss of appetite? Sequencing of the ballan wrasse (Labrus bergylta) genome and intestinal transcriptomic profiling illuminate the evolution of loss of stomach function in fish
Source: BMC Genomics. 2018 Mar 6;19:186. doi: 10.1186/s12864-018-4570-8 (PMC5840709; doi:10.1186/s12864-018-4570-8)
Supplement: Supplementary file 2 — Data showing full length analysis of the Illumina de novo brain and intestine transcriptome assembly using BLAST+ and Uniprot as a reference database (release). The metric analysis examines the number of assembled transcripts that appeared to be full length or nearly full length. The first column (#hit_pct_cov_bin %) shows the present coverage in the database. The second column (count_in_bin) shows the number proteins that match a trinity assembly transcript by more than the present given in the table >%. E.g 1554 proteins match a transcript by > 80% and <= 90% of their protein lengths. The third column (>bin_below) shows the number of protein in the reference database that are represented by nearly full length transcripts with a % alignment coverage. (PDF 285 kb) [file 12864_2018_4570_MOESM2_ESM.pdf]

Full length analysis of the Illumina de novo brain and intestine transcriptome assembly

#hit_pct_cov_bin %	count_in_bin	>bin_below
**100**	7730	7730
**90**	1554	9284
**80**	1013	10297
**70**	812	11109
**60**	844	11953
**50**	790	12743
**40**	704	13447
**30**	668	14115
**20**	535	14650
**10**	182	14832
