# Supplementary figures and images for: Loss of stomach, loss of appetite? Sequencing of the ballan wrasse (Labrus bergylta) genome and intestinal transcriptomic profiling illuminate the evolution of loss of stomach function in fish
Source: BMC Genomics. 2018 Mar 6;19:186. doi: 10.1186/s12864-018-4570-8 (PMC5840709; doi:10.1186/s12864-018-4570-8)

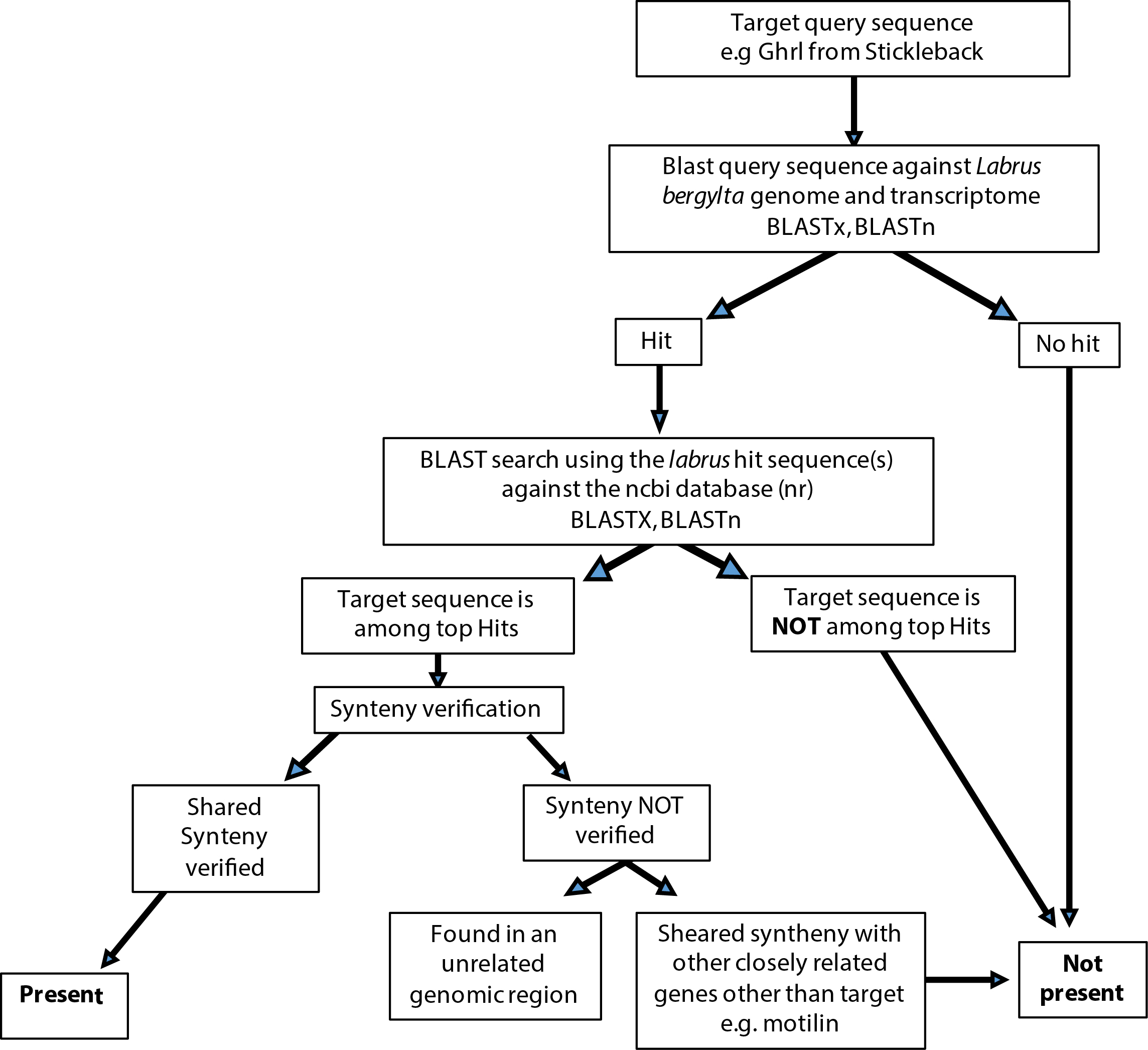

Supplement: Supplementary file 3 — Schematic figure showing the strategy for gene search and for deciding whether a gene is present (“Present”) in the wrasse genome or not (“Not present”). (PNG 322 kb) [file 12864_2018_4570_MOESM3_ESM.png]

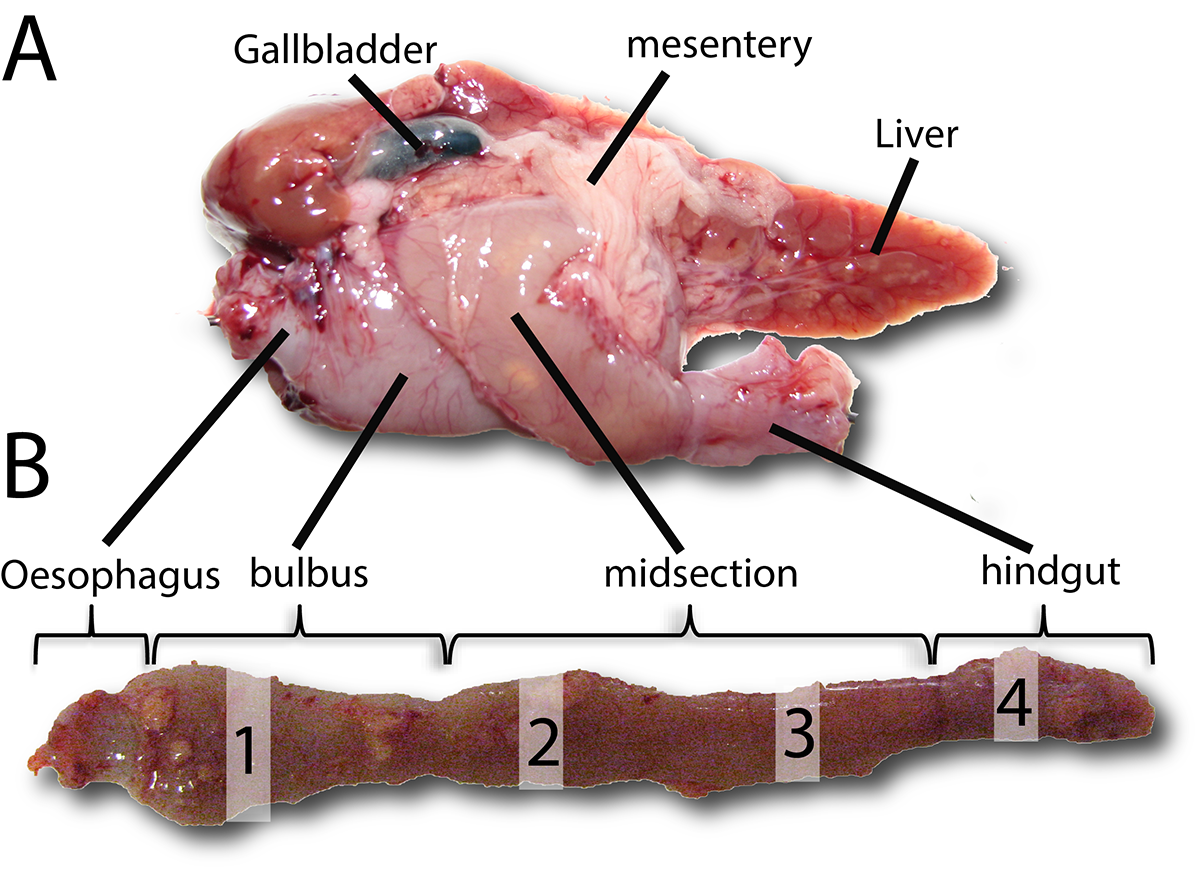

Supplement: Supplementary file 5 — Figure showing the digestive organs of the Ballan wrasse and a schematic depiction of the collected intestinal segments. (TIFF 3090 kb) [file 12864_2018_4570_MOESM5_ESM.tif]
