# Supplementary material for: Loss of stomach, loss of appetite? Sequencing of the ballan wrasse (Labrus bergylta) genome and intestinal transcriptomic profiling illuminate the evolution of loss of stomach function in fish
Source: BMC Genomics. 2018 Mar 6;19:186. doi: 10.1186/s12864-018-4570-8 (PMC5840709; doi:10.1186/s12864-018-4570-8)
Supplement: Supplementary file 4 — The figures shows the genomic region with Ensembl coordinates and flanking genes (Synteny) for mboat4, motilin receptor (mlnr), peptide y a (pyya), peptide y b (pyyb), cholecystokinin a (ccka), cholecystokinin b (cckb). (PDF 333 kb) [file 12864_2018_4570_MOESM4_ESM.pdf]

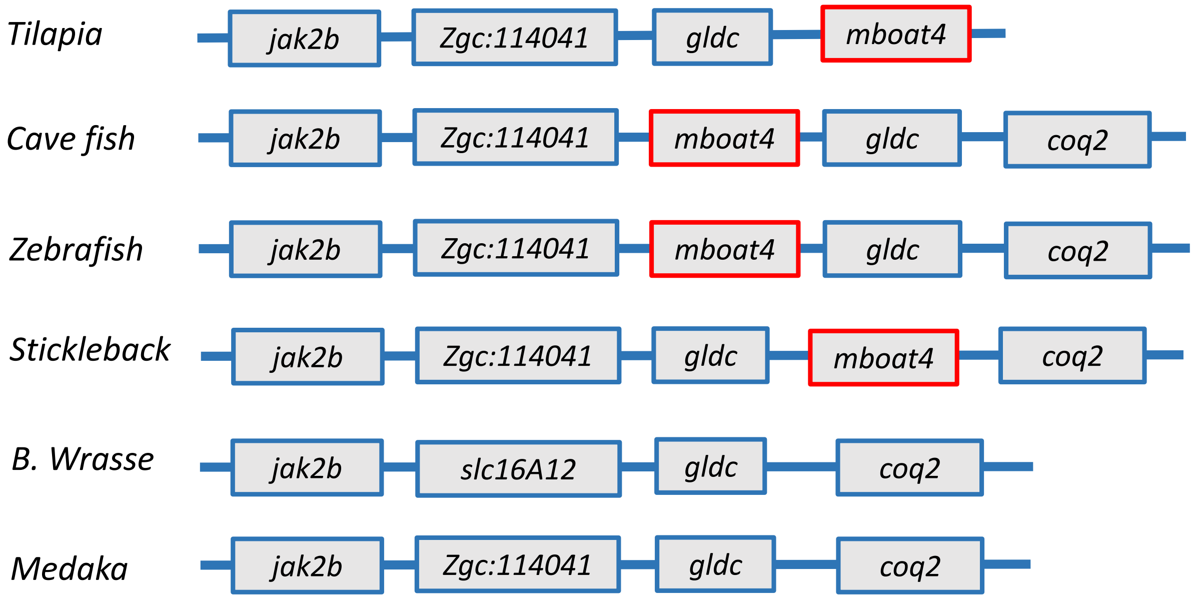

Figure A. Genomic region and Ensembl accession number for mboat4 genes in teleosts. Tilapia (*Oryzias latipes*) GL831307.1:236952-311998, (Gene not present in the Ensembl gene prediction. sequences identified in the present work in the given genomic region by tBLASTn search. ENSDART00000159371 was used as query. e-value = 2e-65); Cave fish (*Astyanax mexicanus*): KB882180.1:726758-827132, ENSAMXG00000014423; Stickleback (*Gasterosteus aculeatus*): groupXIII:17231488-17271128, ENSGACG00000013734; Zebrafish (*Danio rerio*): chromosome 5:65362599-65538959, ENSDARG00000018882; Ballan wrasse (*Labrus bergylta*): LaB_20160104_scaffold_208:294158-362772; Medaka (*Oryzias latipes*): chromosome 9:3938996-4019818, ENSORLG00000001556

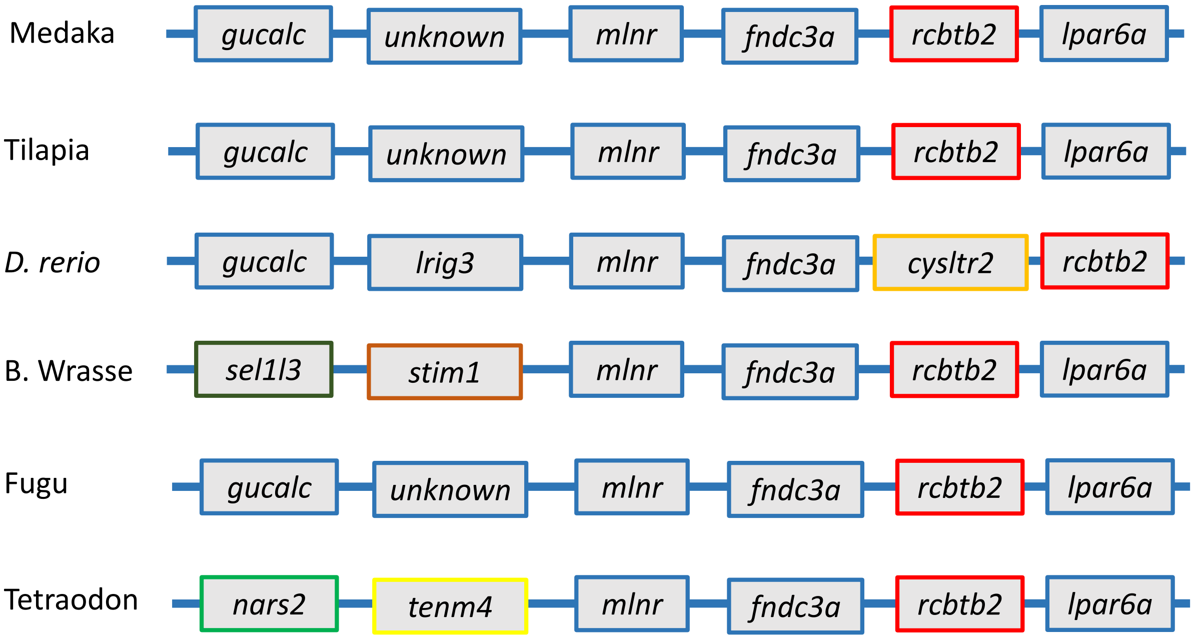

Figure B. Genomic region and Ensembl accession number for motilin receptor (*mlnr*) in teleosts. Tilapia (*Oryzias latipes*) (GL831218.1:1557919-1666013) ENSONIG00000005699; zebrafish (*Danio rerio*): 15:2925420-3178148, ENSDARG00000091207; Ballan wrasse (*Labrus bergylta*): LaB_20160104_scaffold_254:674,888-784,422, LABE_00040338; Tetraodon (*Tetraodon nigroviridis*): 16:5671222-5876201, ENSTNIG00000019039; Fugu (*Takifugu rubripes*) scaffold_19:1379444-1441687, ENSTRUG00000013958; Tilapia GL831218.1:1556244-1666009, ENSONIG00000005699.

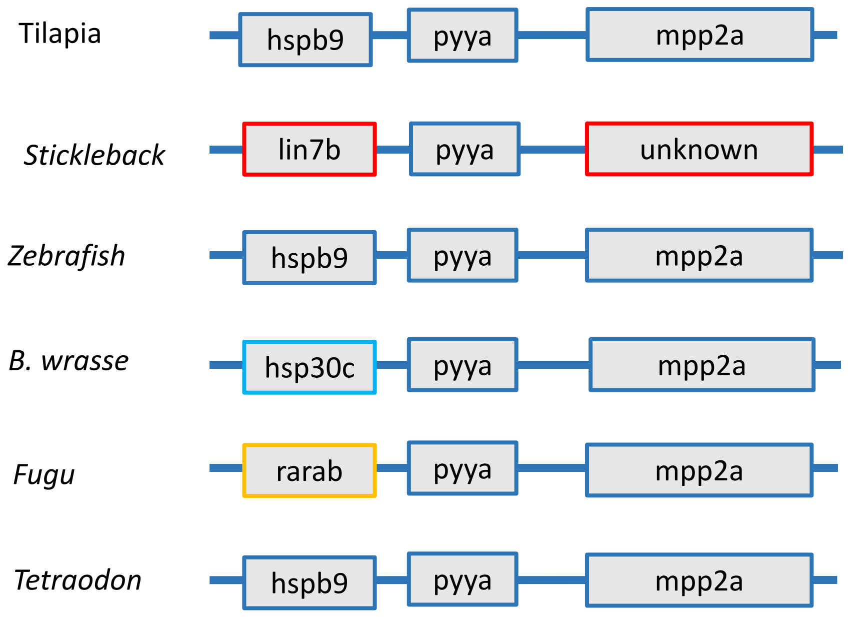

Figure C. Genomic region and Ensembl accession number for peptide y a (*pyya*) in Tilapia (*Oreochromis niloticus*) GL831179.1:1232875-1317458, ENSONIG00000006306; Stickleback (*Gasterosteus aculeatus*) scaffold_80:221815-302071, ENSGACG00000000462; Zefbrafish (*Danio rerio*) 3:33168172-33252755, ENSDARG00000053449; Ballan wrasse (*Labrus bergylta*) 160104_scaffold_338:468,307-515,344; Fugu (*Takifugu rubripes*) scaffold_431:13575-110693, ENSTRUG00000010705; Tetraodon (*Teraodon nigroveridis*) Un_random:4460723-4485961, ENSTNIG00000011155.

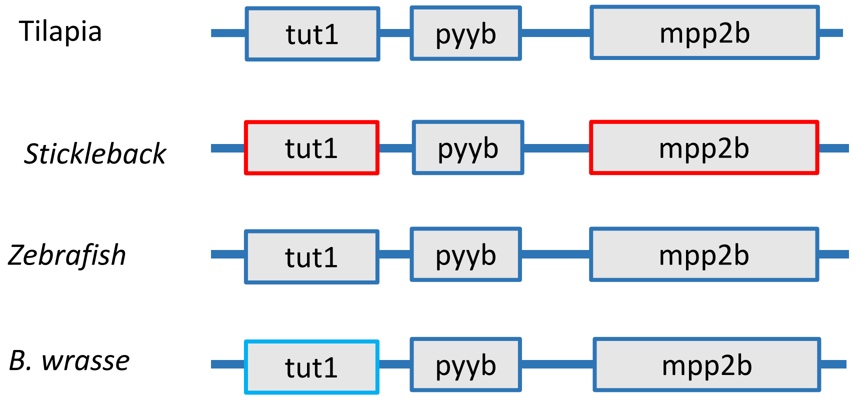

Figure D. Genomic region and Ensembl accession number for peptide y b (*pyyb*) in Tilapia (*Oreochromis niloticus*) GL831136.1:7361496-7437894, ENSONIG00000019912; Stickleback (*Gasterosteus aculeatus*) scaffold_80:221815-302071, ENSGACG00000009947; Zefbrafish (*Danio rerio*) 12:10160367-10315209, ENSDARG00000035832; Ballan wrasse (*Labrus bergylta*) LaB_20160104_scaffold_398:85,254-162,953; Fugu (*Takifugu rubripes*) scaffold_431:13575-110693, ENSTRUG00000016462; Tetraodon (*Teraodon nigroveridis*) Un_random:4460723-4485961, ENSTNIG00000001330.

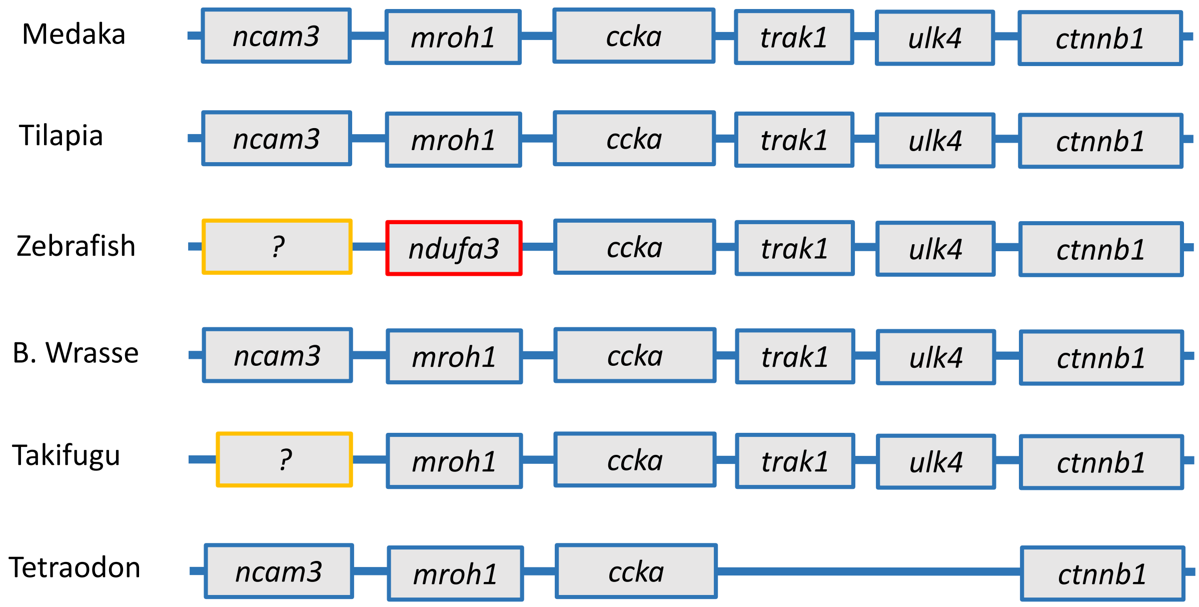

Figure E. Genomic region and Ensembl accession number for cholecystokinin a (*ccka*) gene in teleosts. Medaka (*Oryzias latipes*): Chromosome 16:11129168-11331760, ENSORLG00000005949; Tilapia (*Oreochromis niloticus*): GL831158.1:3480909-3755124, ENSONIG00000007246; Ballan wrasse (*Labrus berggylta)*: LaB_20160104_scaffold_3:3,768,618-3,898,146, LABE_00001570; (*Danio rerio*):16:5749306-6317598, ENSDARG00000070810; Fugu (*Takifugu rubripes*): scaffold_383:132-105503, ENSTRUG00000001951; Tetraodon (*Tetraodon nigroviridis*): chromosome 8:3356248-3456179, ENSTNIG00000019093.

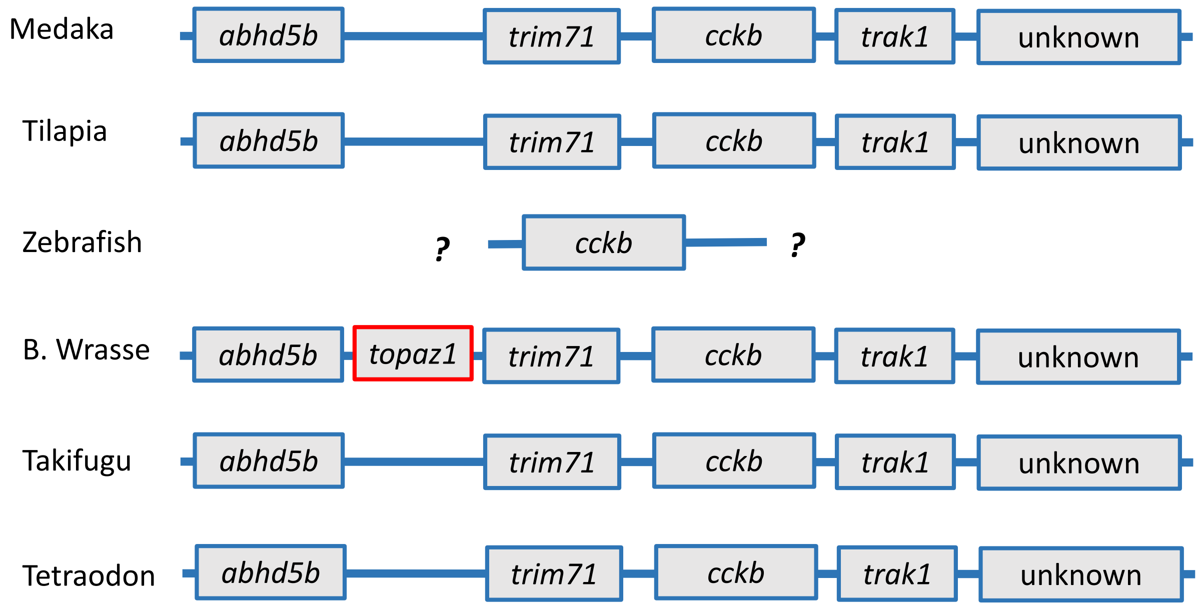

Figure F. Genomic region and Ensembl accession number for cholecystokinin b (*cckb*) gene in: Medaka (*Oryzias latipes*): Chromosome 11:13403405-13610771, ENSORLG00000005594; Tilapia (*Oreochromis niloticus*): GL831437.1:387788-564545, ENSONIG00000015434; Ballan wrasse (*Labrus berggylta)*: LaB_20160104_scaffold_422:1-129,305, LABE_00051154; (*Danio rerio*):KN150698.1:1521-8568, ENSDARG00000070810; Fugu (*Takifugu rubripes*): scaffold_12:826058-910300, ENSTRUG00000013433; Tetraodon (*Tetraodon nigroviridis*):
