# Supplementary material for: Loss of stomach, loss of appetite? Sequencing of the ballan wrasse (Labrus bergylta) genome and intestinal transcriptomic profiling illuminate the evolution of loss of stomach function in fish
Source: BMC Genomics. 2018 Mar 6;19:186. doi: 10.1186/s12864-018-4570-8 (PMC5840709; doi:10.1186/s12864-018-4570-8)
Supplement: Supplementary file 8 — Clustering of gene ontology terms (GOs) enriched in segment 1 (Fig. A) and segment 4 (Fig. B). REVIGO summarizing and visualizing software [90] was used to construct a tree map clustering of gene ontology terms (GO) enriched in the hind gut (segment 1) of ballan wrasse intestine using. Box size indicate the relative size of the GO cluster. Colors are added to separate the main hierarchy GO terms. The figure indicates the largest enriched clusters of GO terms in segment 1 and 4. (PDF 443 kb) [file 12864_2018_4570_MOESM8_ESM.pdf]

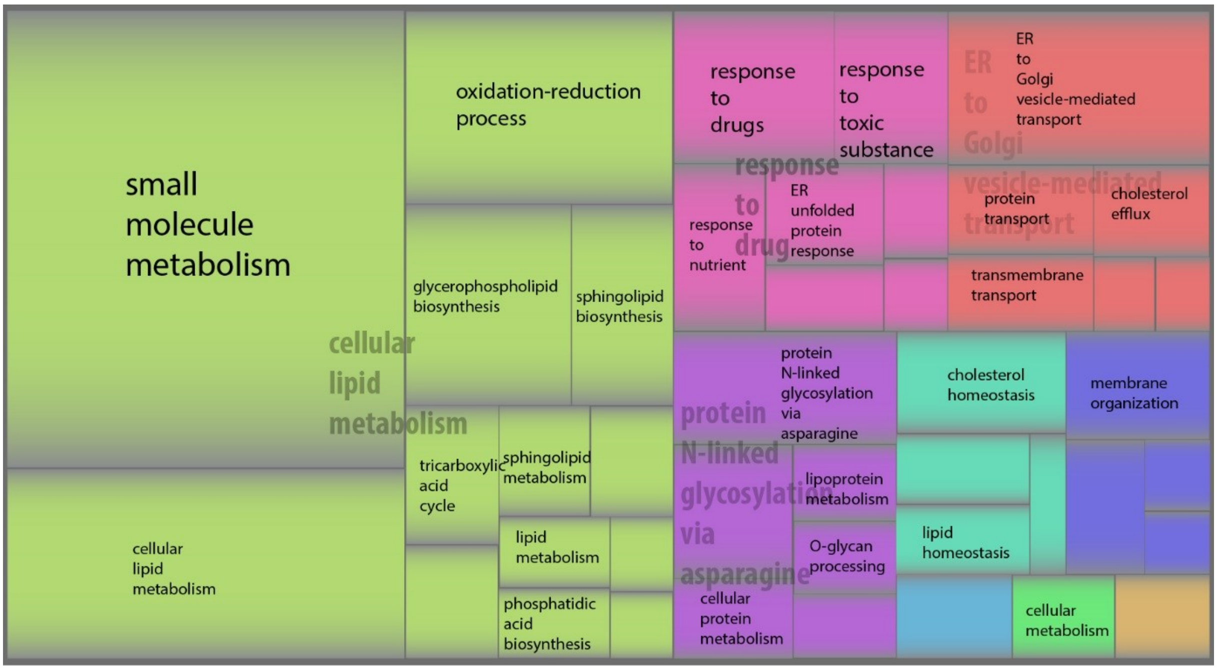

A. Clustering of gene ontology terms (GOs) enriched in segment 1. REVIGO summarizing and visualizing software [9] was used to construct a tree map clustering of gene ontology terms (GO) enriched in the hind gut (segment 1) of ballan wrasse intestine using. The figure indicates the largest clusters of GO terms enriched in the anterior gut (segment 1 vs 4)

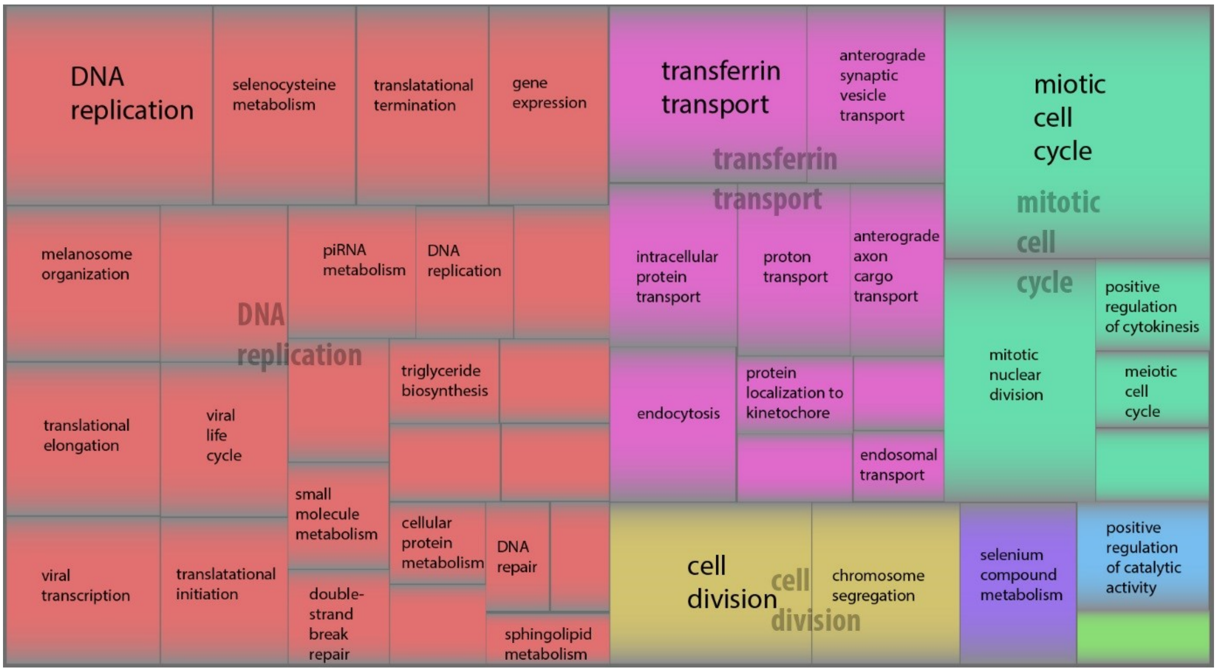

B. Clustering of gene ontology terms (GOs) enriched in segment 4. REVIGO summarizing and visualizing software [9] was used to construct a tree map clustering of gene ontology terms (GO) enriched in the hind gut (segment 4) of ballan wrasse intestine using. The figure indicates the largest clusters of GO terms enriched in the anterior gut (segment 4 vs 1)
