# Supplementary material for: Loss of stomach, loss of appetite? Sequencing of the ballan wrasse (Labrus bergylta) genome and intestinal transcriptomic profiling illuminate the evolution of loss of stomach function in fish
Source: BMC Genomics. 2018 Mar 6;19:186. doi: 10.1186/s12864-018-4570-8 (PMC5840709; doi:10.1186/s12864-018-4570-8)
Supplement: Supplementary file 9 — Top ten differentially expressed genes based on fold change (positive or negative) between segment 1 and 4 (Upregulated and downregulated genes in segment 1 compared to 4. The table shows Gene_ID, gene_symbol, Fold change and p-adjust values. (PDF 258 kb) [file 12864_2018_4570_MOESM9_ESM.pdf]

Differentially expressed genes in segment 4 versus 1.

| *Gene_ID* | *gene_symbol* | *enriched* | *FoldChange* | *p-adj* |
|---|---|---|---|---|
| LABE_00025543 | *cubn* | Segment 4 | 416 | 1.38E-182 |
| LABE_00002019 | *lrp2* | Segment 4 | 362 | 2.65E-172 |
| LABE_00048734 | *mrc1* | Segment 4 | 362 | 5.81E-165 |
| LABE_00042575 | *ctsb* | Segment 4 | 274 | 5.28E-192 |
| LABE_00047336 | *p2rx5* | Segment 4 | 169 | 1.10E-90 |
| LABE_00033481 | *ctsl* | Segment 4 | 137 | 1.56E-104 |
| LABE_00035304 | *unknown* | Segment 4 | 104 | 9.95E-84 |
| LABE_00070741 | *sh3tc1* | Segment 4 | 97 | 4.99E-52 |
| LABE_00003530 | *amn* | Segment 4 | 84 | 5.82E-89 |
| LABE_00007510 | *unknown* | Segment 4 | 79 | 2.44E-36 |
| LABE_00047478 | *Cyp2j-like* | Segment 1 | -11 | 3.95E-25 |
| LABE_00077722 | *dgka* | Segment 1 | -11 | 1.63E-13 |
| LABE_00068071 | *tshr* | Segment 1 | -12 | 6.18E-11 |
| LABE_00065259 | *slc25a22* | Segment 1 | -12 | 9.93E-17 |
| LABE_00033114 | *rhbg* | Segment 1 | -13 | 1.37E-12 |
| LABE_00006063 | *gcsh* | Segment 1 | -13 | 2.68E-18 |
| LABE_00029363 | *cyp2j-like* | Segment 1 | -15 | 6.56E-25 |
| LABE_00009863 | *etnppl* | Segment 1 | -16 | 4.85E-21 |
| LABE_00033129 | *dgka* | Segment 1 | -18 | 3.04E-24 |
| LABE_00046718 | *pyya* | Segment 1 | -34 | 3.16E-29 |
